# microRNAs Sculpt Neuronal Communication in a Tight Balance That Is Lost in Neurological Disease

**DOI:** 10.3389/fnmol.2018.00455

**Published:** 2018-12-12

**Authors:** Kristen T. Thomas, Christina Gross, Gary J. Bassell

**Affiliations:** ^1^Department of Developmental Neurobiology, St. Jude Children’s Research Hospital, Memphis, TN, United States; ^2^Division of Neurology, Cincinnati Children’s Hospital Medical Center, Cincinnati, OH, United States; ^3^Department of Pediatrics, College of Medicine, University of Cincinnati, Cincinnati, OH, United States; ^4^Department of Cell Biology, Emory University School of Medicine, Atlanta, GA, United States; ^5^Department of Neurology, Emory University School of Medicine, Atlanta, GA, United States

**Keywords:** microRNA, neuronal signal transduction, miR-137, epilepsy, schizophrenia, miRNA biogenesis, BDNF, Nrg1

## Abstract

Since the discovery of the first microRNA 25 years ago, microRNAs (miRNAs) have emerged as critical regulators of gene expression within the mammalian brain. miRNAs are small non-coding RNAs that direct the RNA induced silencing complex to complementary sites on mRNA targets, leading to translational repression and/or mRNA degradation. Within the brain, intra- and extracellular signaling events tune the levels and activities of miRNAs to suit the needs of individual neurons under changing cellular contexts. Conversely, miRNAs shape neuronal communication by regulating the synthesis of proteins that mediate synaptic transmission and other forms of neuronal signaling. Several miRNAs have been shown to be critical for brain function regulating, for example, enduring forms of synaptic plasticity and dendritic morphology. Deficits in miRNA biogenesis have been linked to neurological deficits in humans, and widespread changes in miRNA levels occur in epilepsy, traumatic brain injury, and in response to less dramatic brain insults in rodent models. Manipulation of certain miRNAs can also alter the representation and progression of some of these disorders in rodent models. Recently, microdeletions encompassing *MIR137HG*, the host gene which encodes the miRNA miR-137, have been linked to autism and intellectual disability, and genome wide association studies have linked this locus to schizophrenia. Recent studies have demonstrated that miR-137 regulates several forms of synaptic plasticity as well as signaling cascades thought to be aberrant in schizophrenia. Together, these studies suggest a mechanism by which miRNA dysregulation might contribute to psychiatric disease and highlight the power of miRNAs to influence the human brain by sculpting communication between neurons.

## Introduction

The human brain contains over 100 billion neurons, which together mediate its diverse functions including perception, motor control, learning and memory, and even consciousness. Each of these functions requires rapid communication between neurons in distant brain regions. Much of this communication involves neurotransmitter signaling at the estimated 100 trillion synaptic connections in the human brain, and this classical synaptic activity is further modulated by growth factors and other signals at synaptic and extra-synaptic sites. Many of these events shape, and are shaped by, the activity of small non-coding RNAs known as microRNAs (miRNAs).

## miRNAs: History and Significance for Neural Biology

**FIGURE 1:**
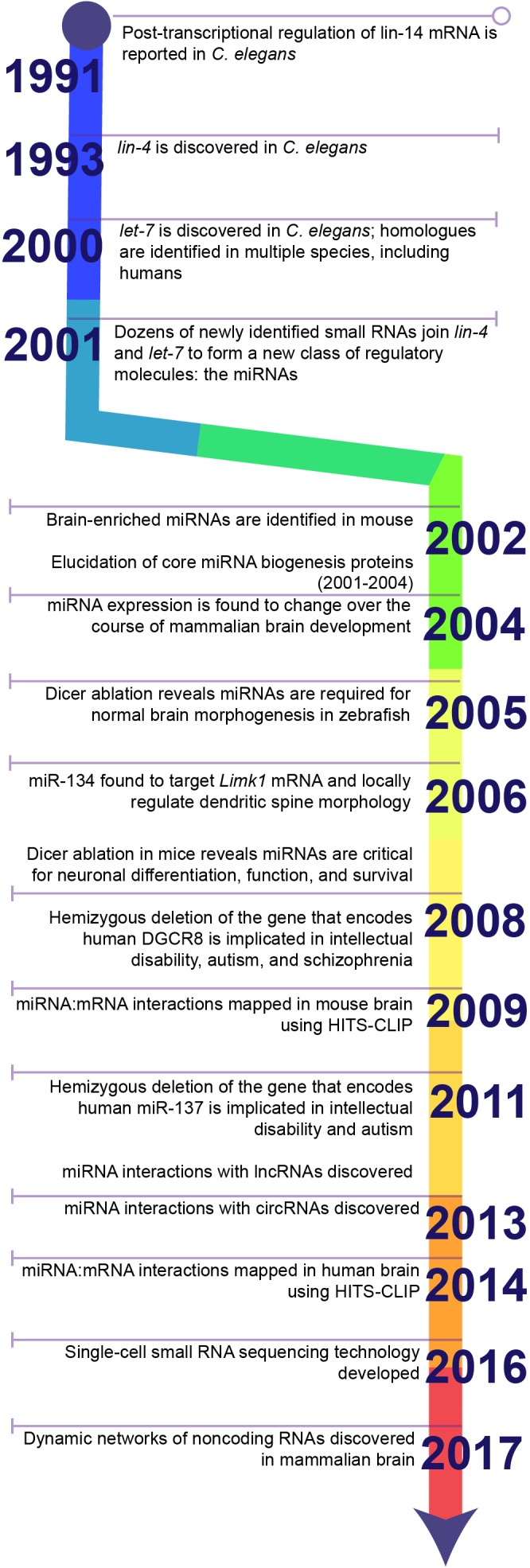
Timeline highlighting key discoveries in the history of miRNA research. The discovery of 3′UTR-dependent regulation of lin-14 translation in C. elegans paved the way for the discovery of the first miRNA: lin-4. Failure to discover homologues of lin-4 in other model systems, e.g., Drosophila or rodents, or in humans stalled the progression of miRNA research until the discovery of a second miRNA, let-7, and its homologues. Sequencing studies soon identified dozens of additional miRNAs in a wide diversity of species and established miRNAs as a conserved mechanism for post-transcriptional regulation of gene expression. Later studies demonstrated that miRNAs are critical for brain development and neuronal function. Ongoing research in the last decade has greatly expanded the toolkit for examining miRNA biology and continues to uncover novel functions for miRNAs within the brain.

In 1991, Gary Ruvkun’s lab reported an unusual observation from *Caenorhabditis elegans*: deletion of two small sequences in the 3′untranslated region (3′UTR) of the *lin-14* mRNA caused *lin-14* protein to accumulate (Wightman et al., 1991) (Figure 1). These deletions did not affect the stability or function of *lin-14* protein, suggesting they must function in post-transcriptional regulation of *lin-14* mRNA. The authors hypothesized that an unidentified regulatory factor must bind to these sequences and repress the synthesis of the encoded protein. Two years later, the source of this translational repression was identified and became the first in a new class of regulatory molecules: the miRNAs.

In 1993, Victor Ambros’s lab reported that the *lin-4* locus in *C. elegans* gives rise to two small RNA products: one of 22 nucleotides and one of 61 nucleotides (Lee et al., 1993), both of which contain sequences complementary to seven repeated sequences that lie within the *lin-14*-3’UTR and that repress *lin-14* protein levels (Wightman et al., 1993). In two separate articles within the same issue of *Cell*, Ambros’s and Ruvkun’s groups proposed a model in which *lin-4* small RNAs bind to the *lin-14*-3′UTR and repress mRNA translation (Lee et al., 1993; Wightman et al., 1993). Later research would reveal that the 61 nucleotide RNA is a precursor for the functional 22 nucleotide *lin-4* RNA that binds the *lin-14-*3’UTR. Unfortunately, however, *lin-4* had no apparent homologues in *Drosophila* or mammalian model systems, so the significance of this discovery would not be fully appreciated for some years.

In 2000, Ruvkun’s lab reported that another small RNA, which they called *let-7*, targets complementary regulatory sequences in the 3’UTRs of multiple mRNAs in *C. elegans*, including *lin-14* (Reinhart et al., 2000). Unlike *lin-4*, however, homologues to *let-7* were identified in a range of animal species, including *Drosophila* and humans (Pasquinelli et al., 2000). These small RNAs were no longer an oddity only observed in worms, but a conserved regulatory mechanism that might control human gene expression.

In 2001, three separate reports in a single issue of *Science* identified a large class of small 19-24 nucleotide non-coding RNAs present in *C. elegans, Drosophila melanogaster*, and *Dictyostelium discoideum* (Lagos-Quintana et al., 2001; Lau et al., 2001; Lee and Ambros, 2001). These small RNAs were generated from larger 60–70 nucleotide precursors that formed stem loop structures through complementary base pairing within the precursor sequence (Hutvágner et al., 2001). Like *let-7*, several were highly conserved across species. The authors collectively referred to these small RNAs as microRNAs, or miRNAs, and proposed based on their similarity to *let-7*and *lin-4* that they form a new class of regulatory molecule that binds to target mRNAs through complementary base pairing and inhibits translation.

Afterward, the pace of miRNA research accelerated rapidly. The proteins that mediate miRNA synthesis were identified in parallel with the elucidation of the RNA interference (RNAi) pathway. In 2001, Dicer was identified as the enzyme that generates mature miRNAs by cleaving double-stranded miRNA precursors (Hutvágner et al., 2001; Martinez et al., 2002). Later studies identified Drosha as the enzyme that generates miRNA precursors from longer primary transcripts (Lee et al., 2003) and the Argonaute proteins (Ago1-4 in humans) that directly bind to miRNAs and help mediate their effects on mRNA targets (Martinez et al., 2002; Liu et al., 2004; Meister et al., 2004; Pillai et al., 2004; Rand et al., 2004). Ongoing research continues to provide insights into the intricate molecular mechanisms that govern miRNA synthesis.

Soon it became clear that miRNAs play a critical role in the development and function of the central nervous system. Analysis of mouse tissues revealed a number of brain-enriched miRNAs (Lagos-Quintana et al., 2002). Krichevsky et al. (2004) and Miska et al. (2004) found that miRNA expression was differentially regulated over the course of mammalian brain development, and Kim et al. (2004). identified over 80 miRNAs expressed in mammalian neurons. Disruption of Dicer revealed that miRNAs are critical for brain morphogenesis in zebrafish (Giraldez et al., 2005). The expression of a subset of miRNAs was also found to be induced by neuronal differentiation from embryonal carcinoma cells (Sempere et al., 2004). These early studies collectively suggested that miRNAs regulate brain development and neuronal function.

In a landmark publication, Schratt et al. (2006) provided the first evidence of a specific miRNA-target pair that regulates synaptic function. In cultured neurons, miR-134 localizes to the postsynaptic compartment and locally regulates the translation of *Limk1* mRNA, which encodes a kinase critical for dendritic spine development. miR-134 overexpression reduces the size of dendritic spines, but restoration of Limk1 rescues spine morphology. miR-134, therefore, limits dendritic spine size by inhibiting the translation of *Limk1* mRNA, demonstrating that even single miRNAs may significantly impact the neural circuitry. A role of miR-134 in dendritic spine morphology and the regulation of neuronal excitability through *Limk1* mRNA was later corroborated *in vivo* (Jimenez-Mateos et al., 2012, 2015).

Ongoing research continues to demonstrate that miRNAs play a significant role in neural biology. A series of studies in 2007 and 2008 used conditional Dicer ablation to demonstrate that miRNA depletion in neuronal progenitors and in subpopulations of mature neurons leads to impaired neuronal differentiation and function and, in some cases, to neurodegeneration in the mammalian brain (Kim et al., 2007; Schaefer et al., 2007; Cuellar et al., 2008; Davis et al., 2008; De Pietri Tonelli et al., 2008). Hemizygous deletions affecting individual miRNAs or miRNA pathway components later demonstrated that miRNA depletion could cause neurological deficits in human patients (Stark et al., 2008; Willemsen et al., 2011).

Techniques to examine miRNAs have expanded greatly within the last decade. High throughput RNA sequencing combined with crosslinking immunoprecipitation (HITS-CLIP) has allowed miRNA:mRNA interactions to be comprehensively mapped within mouse and human brain (Chi et al., 2009; Boudreau et al., 2014). Single-cell small RNA sequencing technology allows us to examine the diversity of miRNAs within a single cell (Faridani et al., 2016). New miRNAs and miRNA:mRNA target interactions continue to be identified within the mammalian nervous system, and each expands our understanding of the significance of miRNAs for neural biology. In addition to mRNA targeting, recent research has revealed that miRNA target other non-coding RNAs, e.g., long non-coding RNAs and circular RNAs, and that these interactions regulate neuronal development and function (Cesana et al., 2011; Hansen et al., 2013; Rani et al., 2016; Piwecka et al., 2017; Kleaveland et al., 2018).

miRNAs are also highly dynamic species that can shape the way the cell responds to changes in its environment, and few cells respond as dramatically to these changes as the neuron. In the sections that follow, we will summarize our current understanding of the complex relationship between miRNAs and various forms of neuronal signaling. We will begin with the miRNA biogenesis pathway and the mechanisms by which neuronal signaling regulates this pathway. We will then discuss the mechanisms by which miRNAs tune neuronal signaling events that are critical for neuronal development, synaptic transmission, and synaptic plasticity. Finally, we will examine in detail miR-137 and the mounting evidence that suggests that this miRNA plays intriguing roles in human brain function and that its dysregulation contributes to psychiatric disease.

## The miRNA Pathway Is Tightly Regulated in Neurons

**FIGURE 2:**
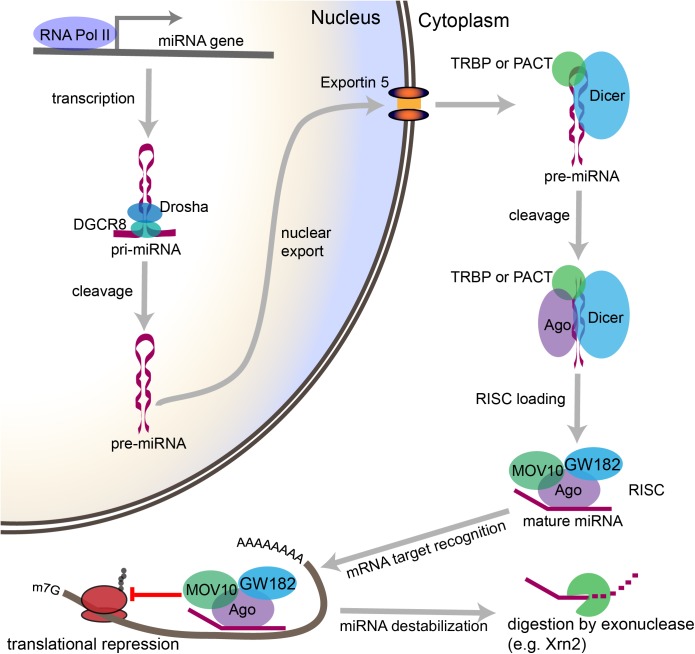
Overview of the canonical miRNA pathway, from synthesis to degradation. miRNA synthesis begins with transcription of a miRNA-encoding gene, which may lie within a protein coding gene or within the intergenic space, to form a primary miRNA (pri-miRNA). Within the nucleus, stem loop structures in the pri-miRNA are recognized by DGCR8, which then recruits the enzyme Drosha to cleave the pri-miRNA at the base of the stem loop to form a precursor miRNA (pre-miRNA). The pre-miRNA is then exported from the nucleus through Exportin-5. In the cytoplasm, the pre-miRNA is recognized by a complex containing Dicer and either TRBP or PACT. Dicer cleaves the loop structure from the pre-miRNA to form a double stranded structure. One of the two strands is then loaded into an Ago-containing complex to form the RNA-induced silencing complex (RISC), and the second strand is degraded. The miRNA then acts as a guide, which allows the RISC to recognize mRNAs containing complementary sequences. Once the miRNA binds the mRNA target sequence, the protein components of the RISC, particularly GW182, repress the translation of the mRNA. Modifications to the 3′ end of the miRNA can stabilize or destabilize the miRNA. The lifecycle of the miRNA ends with digestion by exonucleases.

**FIGURE 3:**
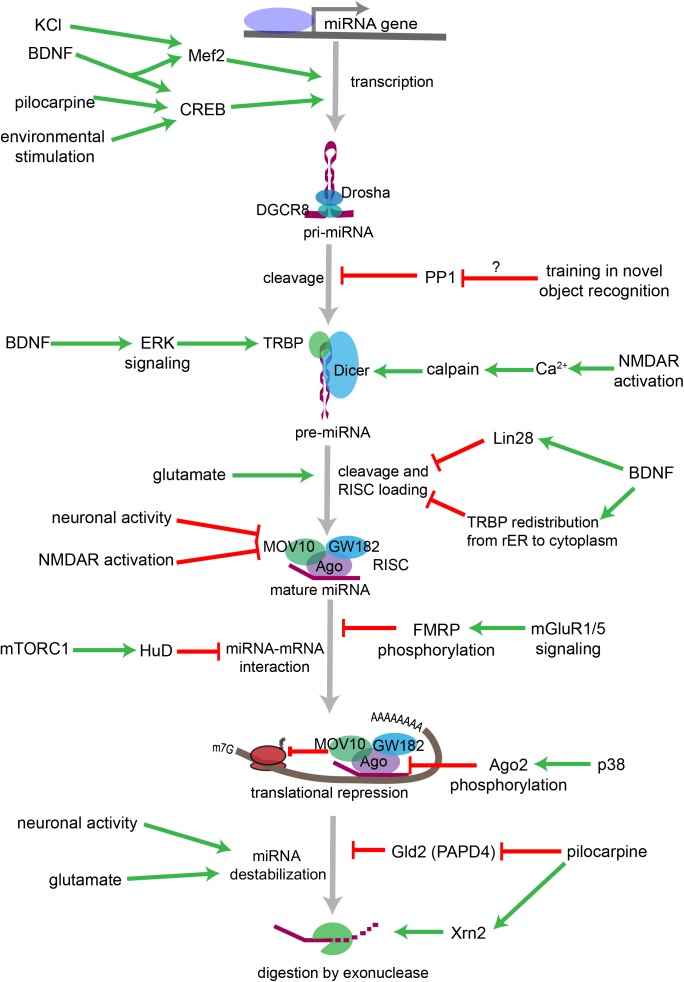
Overview of neuronal signaling events that influence miRNA biogenesis, activity, and degradation. Each step within the miRNA biogenesis pathway may be stimulated (green arrow) or inhibited (red bar) by intra- and extracellular signaling events. Pri-miRNA levels increase when BDNF or other signals activate transcription factors that stimulate the transcription of miRNA-encoding genes. Pri-miRNA cleavage by the Microprocessor is influenced by the activity of proteins, such as PP1 which inhibits Microprocessor activity. Pre-miRNA cleavage by Dicer is increased in response to glutamate, BDNF signaling, or NMDA receptor activation. BDNF can also inhibit Dicer’s ability to cleave some pre-miRNAs by inducing Lin28 binding to the pre-miRNA terminal loop or by promoting TRBP redistribution and dissociation from Dicer. Neuronal activity and NMDA receptor activation inhibit RISC activity by promoting the degradation of the RISC component Mov10. miRNA interactions with target mRNAs are also influenced by RNA binding proteins such as FMRP or HuD, which are regulated by gp1 mGluR and mTORC1 signaling, respectively. P38-induced phosphorylation of Ago2 stimulates mRNA translation by causing the RISC to release its bound miRNA. miRNAs may also be destabilized by increases in neuronal activity, by glutamatergic signaling, or by pilocarpine-induced inhibition of the miRNA stabilizing protein Gld2. Pilocarpine may also stimulate miRNA degradation by increasing expression of the exonuclease Xrn2.

miRNA biogenesis refers to the endogenous cellular processes that generate an active miRNA capable of recognizing an mRNA target and regulating its translation (Figure 2). The miRNA biogenesis pathway generates 100s of unique miRNAs in mammalian cells. Each miRNA is capable of targeting 100s if not 1000s of mRNAs. An estimated 70% of mammalian miRNAs are expressed in the brain, and deficits in miRNA biogenesis disrupt neuronal development, function, and survival (Cao et al., 2006; Kim et al., 2007; Schaefer et al., 2007; Cuellar et al., 2008; Davis et al., 2008; De Pietri Tonelli et al., 2008; Hébert et al., 2010). In the sections that follow, we will outline the individual steps within the miRNA biogenesis pathway, the mechanisms by which miRNAs regulate their targets, and the evidence that suggests that regulation of miRNA biogenesis and activity is critical for neuronal function. We will also discuss the mechanisms by which neuronal signaling influences each step of this pathway (Figure 3).

### Within the Nucleus: Transcription and pri-miRNA Processing

miRNA biogenesis begins in the nucleus with the transcription of a miRNA gene to generate the primary miRNA transcript (pri-miRNA). miRNA genes may lie within intergenic space or within the introns or exons of protein coding genes (Rodriguez et al., 2004; O’Carroll and Schaefer, 2013). Many intergenic miRNAs occur within clusters containing multiple miRNA genes within a 0.1–50 kb span, and multiple miRNAs may be transcribed within a single transcript (Lagos-Quintana et al., 2001; Lau et al., 2001). The majority of pri-miRNA transcription is mediated by RNA polymerase II, which also transcribes primary mRNA transcripts, but some pri-miRNAs are transcribed by RNA polymerase III, which transcribes other small RNAs including ribosomal and transfer RNAs (rRNA and tRNA, respectively) (Cai et al., 2004; Lee et al., 2004; Borchert et al., 2006).

In neurons, the transcription factor cAMP-reponse binding protein (CREB) stimulates transcription in response to increased neuronal activity, neurotrophin signaling, and other factors. Vo et al. (2005) identified a CREB binding site upstream of *Mir132* and demonstrated that transcription of the brain-enriched miRNA miR-132 is rapidly induced by brain-derived neurotrophic factor (BDNF) signaling in rat primary cortical neurons. Activation of hippocampal neurons in response to pilocarpine injection or various environmental stimuli also rapidly and robustly increases pri-miR-132 *in vivo* (Nudelman et al., 2010). High frequency stimulation induces transcription of *Mir132* in the adult rat dentate gyrus by an mGluR-dependent mechanism (Wibrand et al., 2010). Similarly, the transcription factor myocyte enhancing factor 2 (Mef2) induces transcription of the miR-379-410 cluster in response to KCl or BDNF stimulation in cultured neurons (Fiore et al., 2009).

During transcription, the pri-miRNA folds into one or more imperfectly paired stem loop structures, which are recognized by the double stranded RNA binding protein DGCR8 (Digeorge syndrome critical region 8). DGCR8 then recruits Drosha, a Ribonuclease III enzyme that catalyzes endonuclease cleavage of the pri-miRNA (Lee et al., 2003; Gregory et al., 2004; Han et al., 2004; Landthaler et al., 2004). Together, Drosha, DGCR8, and associated regulatory proteins form the nuclear protein complex (the Microprocessor complex) that cleaves the pri-miRNA to generate a 60–70 nucleotide precursor miRNA, or pre-miRNA, which contains the characteristic stem-loop structure (Denli et al., 2004; Gregory et al., 2004).

Within the hippocampus, Woldemichael et al. (2016) recently demonstrated that protein phosphatase 1 (PP1) selectively inhibits the processing of the pri-miRNA for miR-182, miR-96, and miR-183. Training on a novel object recognition task significantly enhances miR-182 and miR-183 levels, and both inhibition of PP1 and overexpression of miR-183/96/183 increase long term memory (Woldemichael et al., 2016). However, it is unclear if training inhibits PP1 activity and whether inhibition of PP1 is necessary for memory formation.

Defects in nuclear pri-miRNA processing have been linked to psychiatric disorders. 22q11.2 deletion syndrome (22q11DS), also known as DiGeorge Syndrome, is caused by hemizygous microdeletion of a region on chromosome 22 that encodes the Microprocessor component DGCR8 (Landthaler et al., 2004). Patient phenotypes include intellectual disability and increased risk of autism spectrum disorder (ASD) and attention-deficit/hyperactivity disorder (ADHD), and the majority of 22q11DS patients experience difficulties in adaptive functioning (Biswas and Furniss, 2016). Furthermore, approximately 1 in 4 or 5 patients with 22q11DS will develop schizophrenia. Mouse models for 22q11DS show widespread deficits in mature miRNA levels in the hippocampus (Stark et al., 2008; Earls et al., 2012). DGCR8 deficiency in mice also leads to an age-dependent deficiency in thalamic miR-338-3p, which disrupts thalamic inputs to the auditory cortex (Chun et al., 2014, 2016). Notably, dysregulation of these thalamocortical projections may contribute to auditory hallucinations in schizophrenia patients (Behrendt, 2006; Anticevic et al., 2015). Duplications in the 22q11.2 region are also associated with ASDs but may reduce schizophrenia risk (Rees et al., 2014; Wenger et al., 2016). To our knowledge, the effects of 22q11.2 or *DGCR8* duplication on the miRNA pathway have not been explored, however.

Drosha and DGCR8 also perform functions that are independent of the miRNA pathway, including regulating transcription, mRNA degradation, and DNA repair (Pong and Gullerova, 2018). DGCR8 also performs Drosha-independent functions, such as recruiting the nuclear exosome to degrade small nucleolar RNAs (snoRNAs) (Macias et al., 2012, 2015). The miRNA-independent functions of DGCR8 in particular appear to be critical for cortical development. DGCR8 ablation in apical progenitor cells of the developing cerebral cortex results in less pronounced miRNA loss than Dicer ablation but mediates greater defects in corticogenesis, including reduced cortical thickness and greater structural disorganization (Marinaro et al., 2017). Further studies are needed to determine if these miRNA-independent functions of DGCR8 contribute to neurological deficits in patients with 22q11.2 deletions and duplications.

### Cytoplasmic Processing: The Pre-miRNA

Following Microprocessor-mediated cleavage, the pre-miRNA is exported from the nucleus by Exportin-5 for further processing (Yi et al., 2003; Bohnsack et al., 2004; Lund et al., 2004). In a human non-neuronal cell line, deletion of *XPO5*, which encodes Exportin-5, only modestly affects miRNA levels, suggesting that other nuclear-export mechanisms can compensate for Exportin-5 loss (Kim W. et al., 2014).

Within the cytoplasm, the pre-miRNA undergoes further processing mediated by a protein complex containing Dicer along with TRBP (*trans*-activation response RNA-binding protein) or PACT (protein activator of PKR) (Hutvágner et al., 2001; Chendrimada et al., 2005; Lee et al., 2013). Like Drosha, Dicer is a Ribonuclease III type enzyme, but unlike Drosha, Dicer removes the loop structure from the pre-miRNA to form a double stranded RNA product. One strand of approximately 22 nucleotides from the miRNA duplex becomes a mature, functional miRNA when it is selected for incorporation into the RNA-induced silencing complex (RISC). In most cases, the other strand is degraded. The RISC uses the miRNA as a guide to direct the degradation and/or translational inhibition of mRNAs containing sequences complementary to the miRNA. Within the RISC, the Argonautes are a group of four proteins (Ago1-4) that directly bind to the miRNA and link it to the RISC’s other protein components. Ago2 is the only Ago with known catalytic activity capable of cleaving mRNA targets (Meister et al., 2004).

The thermodynamic properties of the duplex and the sequences of the two strands determine, in part, which strand is selected to be loaded into the RISC (Noland and Doudna, 2013). However, strand selection also differs depending on the cell-type and brain region in which miRNA biogenesis is occurring as well as the developmental stage, and disease state, among other variables that are not well-understood (Lee et al., 2013; Noland and Doudna, 2013). The strand that is incorporated into the RISC is the active form of the miRNA, and the only form with biological activity. Consequently, regulation of the processing of the pre-miRNA to its mature form and the incorporation of the mature miRNA into RISC are critical for the regulation of miRNA activity in neurons and other cell types.

mRNAs that encode proteins that are either secreted or inserted into lipid membranes are translated at the rough endoplasmic reticulum (rER). This suggests that at least a subset of miRNA:mRNA interactions might occur at the rER in neurons and other cell types. Thierfelder et al. (2008) found that mRNAs that encode membrane proteins are over-represented among miRNA targets in HeLa cells. Furthermore, Dicer, TRBP, PACT, and Ago2 associate with the rER in HeLa cells, and the rER appears to be the site of pre-miRNA cleavage, strand selection, and mature miRNA loading into the RISC (Stalder et al., 2013). According to a recent report, the RISC loading complex associates with the rER in the soma and dendrites of developing primary neurons as well (Antoniou et al., 2018). In response to stimulation with BDNF, TRBP dissociates from Dicer and the rER and enters the cytoplasmic space. BDNF signaling modestly affects miRNA levels overall, but significantly reduces the levels of miR-16-5p and increases pre-miR-16, consistent with reduced Dicer activity in the absence of TRBP.

In an alternative model, BDNF increases miRNAs in cultured neurons by increasing TRBP phosphorylation (Huang et al., 2012), which in turn stabilizes TRBP and Dicer and enhances mature miRNA synthesis (Paroo et al., 2009). BDNF also reduces the levels of a subset of miRNAs, the let-7 family of miRNAs, by inducing the expression of the RNA binding protein Lin28, which binds the terminal loop of the pre-miRNA and protects it from Dicer-mediated cleavage (Huang et al., 2012). Additional research regarding the role of BDNF signaling in Dicer-mediated miRNA biogenesis is needed to address discrepancies between the results of these studies (Huang et al., 2012; Antoniou et al., 2018). Both studies agree that the effects of BDNF differ depending on the pre-miRNA in question, and it may be that they also differ according to cellular or developmental context.

In neurons, stimulation of ion channels and neurotransmitter receptors has been shown to alter cytoplasmic microRNA biogenesis. For example, NMDA receptor activity increases dendritic miR-501-3p by a transcription- and miRNA transport-independent mechanism, suggesting that NMDA receptor activity stimulates the Dicer-dependent processing of pre-miR-501 to mature miR-501-3p (Hu et al., 2015).

The elaborate cytoplasmic architecture of neurons adds an additional layer of complexity to these processes. Pre- and mature miRNAs and their associated mRNA targets localize not only to the perinuclear space, but also to distal locations within the elaborately branched axons and dendrites and to the pre- and postsynaptic compartments therein (Lugli et al., 2005, 2008; Sasaki et al., 2014; Kim et al., 2015). For example, within the postsynaptic compartment Dicer associates with the postsynaptic density, leading to the inhibition of Dicer under basal conditions (Lugli et al., 2005). Lugli et al. (2005) proposed a model in which NMDA receptor activity increases intracellular calcium, activating calpain I which in turn enhances synaptic Dicer activity by freeing it from the postsynaptic density, providing an intriguing model for local, activity-induced miRNA maturation.

Pre-miRNA interactions with mRNA binding proteins (mRBPs) also provide a mechanism by which neuronal signaling may regulate pre-miRNA localization. Dendritic targeting of pre-miR-134 is mediated by DEAH-box helicase DHX36 recognizing and binding a specific sequence in the terminal loop of the pre-miRNA (Bicker et al., 2013). BDNF signaling promotes dendritic pre-miR-134 accumulation and dendritic outgrowth in primary neurons, and BDNF-induced dendritic outgrowth is dependent on DHX36 (Zampa et al., 2018). This suggests that BDNF stimulation increases DHX36-dependent transport of pre-miR-134 into dendrites, where pre-miR-134 is then processed to its mature form to locally inhibit the translation of miR-134 targets, e.g., Pumilio 2, that antagonize dendritic outgrowth under basal conditions.

Recently, Sambandan et al. (2017) developed a fluorescent sensor that led to the visualization of miRNA maturation in neuronal dendrites for the first time. The authors observed that using glutamate uncaging to locally stimulate dendrites significantly increased Dicer-dependent cleavage of a pre-miR-181a fluorescent sensor. Furthermore, this stimulation locally reduced the synthesis of a GFP reporter containing a miR-181a target site and reduced the synthesis of endogenous CamKIIα protein, which is encoded by a miR-181a target mRNA. This elegant study demonstrates that neurotransmitter-induced changes in Dicer activity regulate local protein synthesis at postsynaptic sites.

Dicer ablation studies have frequently been used to assess the net effects of miRNA depletion within the brain. However, like Drosha and DGCR8, Dicer performs functions that are independent of the miRNA pathway. Most are thought to take place within the nucleus, including the synthesis of tRNA-derived fragments and the regulation of transcription and DNA repair (Pong and Gullerova, 2018). A recent study claimed that Dicer is an exclusively cytoplasmic protein in mice, and contested the proposed nuclear functions of Dicer (Much et al., 2016). However, Burger and Gullerova (2018) were able to demonstrate using more sensitive techniques (e.g., super-resolution microscopy) that a small fraction (∼5%) of the Dicer protein pool localizes to the nucleus in mouse embryonic fibroblasts. These non-canonical functions of Dicer should be considered when interpreting the results of Dicer ablation studies.

### The Life of a miRNA: Target Interactions, Mechanisms of Action, and Degradation

Within the RISC, miRNAs serve the role of target recognition, i.e., the single miRNA present within the RISC dictates the subset of mRNAs within the cell that are targeted for translational repression. miRNAs recognize their mRNA targets through complementary G/C and A/U base pairing. miRNAs that are perfectly complementary to their mRNA target may induce Ago2-dependent endonucleolytic cleavage of the mRNA, but perfect complementarity is not necessary for target recognition and this mechanism is rarely employed in mammals (Bartel, 2018; Fabian et al., 2010). In mammals, target recognition is usually mediated by complementarity to nucleotides 2-7 of the miRNA, the so-called “seed sequence” (Bartel, 2009). The miRNA target site is, in most cases, located within the 3′ untranslated region (3′UTR) of the target mRNA, though targeting has also been reported in the 5′ untranslated region (5′UTR) and protein coding sequence (CDS) (Easow et al., 2007; Ørom et al., 2008).

After associating with an mRNA target, the protein components of the RISC then mediate the miRNA’s effects on mRNA degradation and/or translation. The Ago proteins bind the miRNA within the RISC and interact with the RISC’s other protein components. Of these, the GW182 family of proteins mediates translational repression (Eulalio et al., 2009). RISC-mediated translational repression may affect the initiation, elongation, or termination stages of translation (Fabian et al., 2010). The mechanism employed in a particular instance of RISC-mediated mRNA regulation may depend on a number of factors, including the particular miRNA, Ago, and GW182 components present, as well as the cell cycle or developmental status of the cell, environmental factors, and the presence or absence of additional RISC regulatory proteins (Fabian et al., 2010).

miRNAs may also destabilize mRNAs. GW182 interactions with the poly(A) binding protein complex (PABPC) recruit the CCR4-NOT1 deadenylase complex to the poly(A) tails of RISC-targeted mRNAs (Behm-Ansmant et al., 2006; Fabian et al., 2009). Deadenylation occurs via the 3′-5′ exoribonuclease activity of the complex. Shortening of the poly(A) tail promotes removal of the 5′ cap of the mRNA, which subsequently leaves the mRNA vulnerable to 5′-3′ exonucleolytic digestion (Chen and Shyu, 2011). Shortening of the poly(A) tail also interferes with translation initiation, so miRNAs may simultaneously inhibit the translation of the mRNA and destabilize the mRNA. However, miRNAs may also repress translation without destabilizing the target mRNA (Filipowicz et al., 2008).

Neuronal activity can stimulate mRNA translation by inducing the degradation of protein components of the RISC. In *Drosophila* neuronal activity stimulates mRNA translation by promoting the degradation of Armitage, an RNA helicase within the RISC that is homologous to mammalian MOV10 (Ashraf et al., 2006). Degradation of Armitage releases RISC targeted mRNAs from miRNA-mediated translational repression and induces synaptic protein synthesis underlying memory formation. Similarly, NMDA receptor activity induces the proteasome-dependent degradation of MOV10 and stimulates dendritic local protein synthesis in murine primary hippocampal neurons (Banerjee et al., 2009). This mechanism may provide a means of specifically stimulating protein synthesis at activated synapses and likely affects all RISC-targeted mRNAs at a given synapse.

The protein components of the RISC may also undergo neuronal signaling-dependent modifications that affect miRNA activity. Phosphorylation of Ago2 regulates miRNA maturation, miRNA guide strand loading, and translational repression (Zeng et al., 2008; Horman et al., 2013; Shen et al., 2013). p38 signaling during neuronal differentiation induces Ago2 phosphorylation at Tyr529, which causes Ago2 to release the miRNA let-7a and stimulates translation of let-7a target mRNAs (Patranabis and Bhattacharyya, 2016). Ago2 phosphorylation or other post-translational modifications induced by neuronal activity have not yet been examined in mature neurons.

A miRNA’s interaction with a particular mRNA target also depends on the presence of competing endogenous RNAs (ceRNAs): RNAs that likewise contain binding sites for the miRNA of interest and compete for binding by that miRNA (Tay et al., 2011). ceRNAs may be other mRNAs, but may also be non-coding RNAs, such as pseudogene transcripts, long non-coding RNAs (lncRNAs), or circular RNAs (circRNAs) (Cesana et al., 2011; Poliseno et al., 2010; Hansen et al., 2013; Kartha and Subramanian, 2014). For example, the primate-specific lncRNA *LncND* contains multiple binding sites for miR-143-3p (Rani et al., 2016). miR-143-3p normally represses Notch signaling by targeting the mRNAs that encode the NOTCH-1 and NOTCH-2 receptors. During human cortical development, high levels of *LncND* in radial glia cells stimulate Notch signaling and promote cell proliferation, even in the presence of high levels of miR-143-3p. Later, declining levels of *LncND* permit miR-143-3p to inhibit Notch signaling, which allows radial glia to differentiate into neurons. Thus, changes in ceRNA levels can promote dynamic cellular processes by influencing miRNA interactions with critical mRNA targets.

Conversely, recent studies suggest that miRNAs influence the activity of other miRNAs that target the same transcripts, and they may interact either synergistically to potentiate miRNA-mediated target repression or antagonistically to block the effects of weaker miRNA interactions (Saetrom et al., 2007; Gam et al., 2018). Therefore, miRNA targeting depends on the sum of the miRNA target sites on a transcript, and the effects of a single miRNA species on an individual target site depend both on the presence of additional miRNA targets sites on that transcript and on the presence of other miRNA species that might bind those sites.

miRNA targeting is also modulated by mRBPs, which may stimulate or inhibit miRNA binding by altering the secondary structure of the target mRNA or competing with miRNAs for a common target site. For example, HuR binding to mRNAs causes the RISC to dissociate from the mRNA, which stabilizes and promotes the translation of the mRNA target (Kundu et al., 2012). Pumilio1 (PUM1) promotes miR-221 and miR-222 binding to the 3′UTR of p27 mRNA by inducing a change in the 3′UTR structure that makes the miRNA target site accessible and represses p27 translation (Kedde et al., 2010). In neurons, HuD and miR-129 compete to regulate Kv1.1 mRNA translation in dendrites (Sosanya et al., 2013). When mTORC1 signaling is active, miR-129 binds to Kv1.1 mRNA and inhibits its translation. When mTORC1 is inhibited by rapamycin, however, HuD binds Kv1.1 mRNA and miR-129 binding is inhibited, thus stimulating Kv1.1 protein synthesis. The regulation of mRNA binding proteins thus provides a mechanism by which neuronal activity can bidirectionally regulate the translation of specific mRNAs.

Furthermore, dysregulation of miRNA-mRBP interactions may contribute to neurodevelopmental disorders, such as Fragile X Syndrome (FXS), which is caused by the loss of the mRBP fragile x mental retardation protein (FMRP). FMRP interacts with protein components of RISC at the genetic and molecular levels (Jin et al., 2004), and FMRP regulates miR-125a-5p interactions with its mRNA target *Dlg4* (Muddashetty et al., 2011). Under basal conditions, FMRP is phosphorylated and inhibits translation by a miR-125a-dependent mechanism. However, when group 1 metabotropic glutamate receptors (mGluR1/5) are activated, FMRP is dephosphorylated and relieves *Dlg4* mRNA of translational repression by miR-125a. Therefore, both miR-125a and FMRP are necessary for this bidirectional regulation of *Dlg4* mRNA translation in neurons. Furthermore, Ago2 association with both miR125a and *Dlg4* mRNA is reduced in synaptoneurosomes from *FMR1* knockout mice (Muddashetty et al., 2011), suggesting FMRP may promote the recruitment and/or stabilization of synaptically localized RISC complexes containing miR-125a and *Dlg4* mRNA. Another study has shown a role for two additional FMRP-associated miRNAs, miR-132 and miR-125b, in altered dendritic spine morphology in FXS (Edbauer et al., 2010). Moreover, the NMDA receptor subunit GluN2A (formerly NR2A) was identified as a target of miR-125b, and GluN2A synthesis was shown to be co-regulated by FMRP and miR-125b. FXS is associated with intellectual disability and increased risk of ASDs and epilepsy, among other neurological symptoms (Tsiouris and Brown, 2004). These symptoms are hypothesized to result from dysregulated translation of FMRP target mRNAs, which may be due in part to dysregulation of miRNA targeting in the absence of FMRP (Weiler et al., 2004; Qin et al., 2005; Muddashetty et al., 2007).

miRNAs are also subject to regulated degradation. These processes remain poorly understood, but recent studies have revealed several underlying principles. First, the number of Ago proteins present may limit the number of mature miRNAs: overexpression of Ago proteins leads to increased levels of mature miRNAs in human cells and, conversely, Ago protein depletion leads to miRNA depletion in *C. elegans* (Grishok et al., 2001; Diederichs and Haber, 2007). Ago proteins appear to stabilize bound miRNAs (Winter and Diederichs, 2011).

Similarly, association with mRNA targets can also stabilize miRNAs, a phenomenon known as TMMP (target-mediated miRNA protection), and the introduction of additional target sites can promote miRNA accumulation (Chatterjee et al., 2011). However, miRNA-mRNA interactions may also destabilize the miRNA and promote its degradation through a process known as targed directed miRNA degradation (TDMD) (Ameres et al., 2010). The effect of the target on miRNA levels appears to be determined by the degree of sequence complementarity, with higher complementarity favoring miRNA degradation and lower complementarity favoring miRNA stabilization.

circRNAs and lncRNAs can regulate miRNA levels through similar mechanisms. A recent study led by Nikolaus Rajewsky’s group demonstrated that the brain-enriched circRNA Cdr1as is a target of miR-671 and miR-7 (Piwecka et al., 2017). Cdr1as contains a single high affinity binding site for miR-671, which limits Cdr1as levels via Ago2-mediated cleavage (Hansen et al., 2011). Cdr1as also contains multiple lower-affinity binding sites for miR-7, through which Cdr1acts may stabilize miR-7 in neurons. In the absence of Cdr1as, miR-7 is downregulated, miR-7 targets are upregulated, and neuronal activity is abnormally increased. By contrast, the lncRNA Cyrano also contains a highly conserved, high-affinity binding site for miR-7 (Ulitsky et al., 2011). Within cerebellar granule neurons, Cyrano promotes miR-7 degradation through TDMD with higher potency than any previously described TDMD mechanism, and each molecule of Cyrano is capable of directing the degradation of multiple miR-7 molecules (Kleaveland et al., 2018). In mice, the loss of Cyrano promotes the accumulation of not only miR-7 but also Cdr1as within the brain, suggesting that these non-coding RNAs form a complex network, with each RNA influencing the levels and/or activity of the others.

The previously described factors that stabilize or destabilize miRNAs are thought to do so by altering the miRNA’s accessibility to enzymes that either (1) modify the ends of the miRNA, or (2) actively degrade the miRNA through exonuclease digestion (Chatterjee and Großhans, 2009; Kai and Pasquinelli, 2010). The poly(A)-polymerase Gld2, for example, stabilizes miRNAs in human fibroblasts by adding a single adenosine residue to the 3′ end of the miRNA (D’Ambrogio et al., 2012). Gld2 also catalyzes monoadenylation of miRNAs within the mouse hippocampus. However, deletion of Gld2 has no detectable effect on miRNA stability in the hippocampus, and the mice exhibit no abnormal behavioral phenotypes (Mansur et al., 2016), suggesting that miRNAs in different tissues and/or species may be differentially regulated by the same enzyme. Other enzymes, e.g., terminal uridyl transferases TUT4 and TUT7, add single uridine residues to the 3’ end of mature miRNAs (Thornton et al., 2014). Uridylation of miR-26b by TUT4 reduces miR-26b’s ability to repress the *IL6*-3′UTR in human A549 cells without affecting miR-26b stability (Jones et al., 2009). Uridylation of the precursor forms of miRNAs, by contrast, has been shown to promote RNA degradation (Heo et al., 2008; Hagan et al., 2009; van Wolfswinkel et al., 2009). miRNA degradation is mediated by exonucleases, such as Xrn1 or Xrn2, and miRNAs that are not stabilized by association with Ago protein or protective mRNA targets are vulnerable to digestion (Chatterjee and Großhans, 2009; Kai and Pasquinelli, 2010).

Kinjo et al. (2016) found that Xrn2 mRNA is increased in the rat hippocampus following status epilepticus, while Gld2 mRNA is reduced. Xrn2 upregulation and Gld2 (also known as PAPD4) downregulation should synergistically promote the degradation of mature miRNAs. However, the effects of status epilepticus on Xrn2 and Gld2 protein levels differed across regions of the hippocampus and across neuronal subtypes. The significance of these findings is currently unclear, and further research is needed to elucidate the mechanisms by which neuronal activity affects miRNA turnover.

Studies examining miRNA degradation have mostly relied on relatively simple model systems, such as cultured cells or *C. elegans*. In most cases, researchers found that only a subset of miRNAs was subject to rapid degradation; however, rapid miRNA turnover in mammalian neurons may be far more prevalent than suggested by other model systems (Krol et al., 2010; Rüegger and Großhans, 2012). Within the retina miRNA turnover is faster in neuronal cells than in non-neuronal cells (Krol et al., 2010). Neuronal activity and glutamatergic signaling significantly increase miRNA turnover and may stimulate the degradation of miRNAs (Krol et al., 2010). Post-mortem studies also suggest that miRNA half-lives may be remarkably short in human brain (∼1–3.5 h) (Sethi and Lukiw, 2009).

Rapid miRNA degradation may be necessary for the rapid induction of mRNA translation that underlies some forms of synaptic plasticity, such as long term potentiation. Conversely, rapid transport and synthesis of miRNAs may also be necessary to inhibit the synthesis of unnecessary proteins under other cellular contexts.

### *In vivo* Stimuli Lead to Differential Expression of miRNAs

miRNA levels in the brain are sensitive to a variety of environmental stimuli, but usually, it is unclear at which step of a miRNA’s life cycle regulation occurs. One dramatic example occurs during episodes of widespread synchronized activity of entire neuronal networks, as seen during seizures. Both acute *status epilepticus* and chronic epilepsy lead to extensive alterations in miRNA levels, as observed in mouse models and in surgical samples from humans with epilepsy (Jimenez-Mateos et al., 2011; Song et al., 2011; Lee et al., 2014; McKiernan et al., 2012). Seizure-evoked miRNA profiles differ depending on the species, the mode of seizure induction, the type of tissue analyzed, and the time of tissue collection after seizure (Henshall, 2014; Korotkov et al., 2017). This suggests that regulation of miRNA levels after seizure is complex and depends on many factors. Similarly to seizures, traumatic brain injury (TBI) alters miRNA profiles in the brains of rat and mouse models (Lei et al., 2009; Redell et al., 2009; Liu L. et al., 2014). Of note, TBI and epilepsy not only induce changes in the brain, but also lead to altered miRNA content of patient blood serum samples (Redell et al., 2010; Wang et al., 2015; Sun et al., 2016; Che et al., 2017). Therefore, miRNAs have been proposed as biomarkers in both diseases, but their clinical utility is not proven yet (Henshall et al., 2016; Martinez and Peplow, 2017; Tiwari et al., 2018).

A caveat of these findings is that different studies analyzing miRNA profiles in epilepsy and TBI rarely identify the same sets of dysregulated miRNAs, even if experimental conditions were very similar (Korotkov et al., 2017; Tiwari et al., 2018). Nevertheless, a few miRNAs have been shown to be dysregulated in multiple epilepsy studies, and some of these miRNAs,, e.g., miR-21 and miR-146a, are also altered in TBI (Aronica et al., 2010; Redell et al., 2011; Harrison et al., 2016; Korotkov et al., 2017). Interestingly, both miR-21 and miR-146a are involved in regulation of neuroinflammation (Bergman et al., 2013; Iori et al., 2017), which is commonly observed in both TBI and epilepsy (Barker-Haliski et al., 2017; Jassam et al., 2017).

miRNA profiles also change after less dramatic brain insults. For example, drugs of abuse such as cocaine (Hollander et al., 2010; Eipper-Mains et al., 2011) as well as environmental toxins (Ji et al., 2018) alter miRNA levels in the brains of mouse models. Whether a similar change can be detected in brain or serum samples of humans is unclear.

Lastly, certain behavioral and learning paradigms have been associated with differential miRNA profiles. Foot shock and other fear conditioning procedures, for example, as well as exposure of mice to the novel object recognition task change the levels of select miRNAs (Lin et al., 2011; Smalheiser et al., 2011; Woldemichael et al., 2016). It is conceivable that even more subtle synaptic plasticity-inducing interventions, such as a novel environment, likewise alter miRNA levels.

So far, it is mostly unknown how the various stages of the miRNA life cycle are impacted by these pathological events or behavioral paradigms. Most likely, regulation occurs at every step from transcription to biogenesis to degradation and differs for specific microRNAs. Overall, although more work is needed to fully understand the underlying mechanisms, these studies suggest that the neuronal plasticity-induced changes in miRNA levels that have been observed following a variety of stimuli may provide a powerful cellular tool to alter neuronal protein levels and, thus, neuronal function.

## miRNAs Play Diverse Roles in the Regulation and Dysregulation of Neuronal Signaling

Just as neuronal signaling events regulate the miRNA pathway, the converse is also true. Recent research suggests that miRNAs regulate neuronal signaling at multiple stages, from the synthesis of pre-synaptic signaling molecules to the transduction of post-synaptic signals. In the following sections, we will highlight the diverse roles of miRNAs in classical neurotransmitter and growth factor signaling as well as in neuronal excitability and intracellular signaling cascades.

### miRNAs in Neurotransmitter Signal Transduction

miRNAs play diverse role in the regulation of neurotransmitter signaling. Neurotransmitters, e.g., glutamate, GABA, dopamine, or serotonin, are synthesized in the presynaptic neuron, packaged into vesicles, and released into the synaptic cleft. They then interact with postsynaptic receptors to stimulate or inhibit the activity of the postsynaptic cell. Each step of the process, from neurotransmitter synthesis to signal termination, depends on the activity of synaptic proteins which may be encoded by mRNAs that are miRNA targets, and some of this regulation can occur locally.

For example, miR-130a and miR-206 synergistically inhibit the synthesis of Substance P in neurons derived from mesenchymal stem cells by targeting *Tac1* mRNA, which encodes the neuropeptide neurotransmitter (Greco and Rameshwar, 2007). The central nervous system utilizes over 100 different neurotransmitters, but to date very few studies have demonstrated a role for miRNAs in the regulation of neurotransmitter synthesis or packaging.

miRNAs regulate the release of neurotransmitter-containing vesicles by targeting mRNAs that encode proteins in the secretory pathway. miR-153 regulates SNAP-25, a core protein of the SNARE complex which mediates calcium induced vesicle fusion with the plasma membrane and the release of neurotransmitter into the synaptic cleft (Wei et al., 2013). Overexpression of miR-153 inhibits the synaptic vesicle cycle at the zebrafish neuromuscular junction, whereas inhibition of miR-153 enhances it. Furthermore, miR-153 activity regulates the movement of zebrafish embryos, presumably by regulating the release of acetylcholine at neuromuscular junctions. Similarly, miR-135a directly targets complexin-1 and -2 (Mannironi et al., 2018), which are important for presynaptic vesicle fusion. Knockdown of miR-135a in the amygdala increases anxiety-like behavior in mice as well as spontaneous excitatory transmission.

Neurotransmitter release is also influenced by miRNAs that regulate presynaptic calcium signaling. miR-25 and miR-185 both target the sarco(endo)plasmic reticulum ATPase SERCA2, which maintains calcium levels in the presynaptic endoplasmic reticulum (Earls et al., 2012). When either miRNA is reduced, such as in 22q11DS model mice, SERCA2 levels are elevated, which in turn elevates calcium levels in the presynaptic cytosol and increases neurotransmitter release.

Following release into the extracellular space, the neurotransmitter binds and activates receptors in the postsynaptic neuron. miRNAs regulate a number of neurotransmitter receptors in the central nervous system, including glutamate, GABA, serotonin, dopamine, and acetylcholine receptors or receptor subunits (Higa et al., 2014). In some cases, a single miRNA may target multiple neurotransmitter receptors with opposing effects on neuronal activity. miR-181a, for example, targets both the AMPA (α-amino-3-hydroxy-5-methyl-4-isoxazolepropionic acid) receptor subunit GluA2 and the GABA_A_α-1 receptor subunit (Saba et al., 2012; Sengupta et al., 2013). Overexpression of miR-181a in primary hippocampal neurons reduces the volume and density of dendritic spines (Saba et al., 2012), suggesting that miR-181a may preferentially inhibit excitatory neurotransmission in this cell type. However, an *in vivo* study demonstrated that inhibition of miR-181a with an antagomir protected against seizure-induced cell death (Ren et al., 2016), suggesting that in other contexts miR-181a exacerbates excitatory neurotransmission. Notably, these studies suggest that knowing a single target of a miRNA may not be sufficient to predict the biological effects of miRNA activity on neuronal signaling.

The signaling effects of many neurotransmitters are terminated by reuptake of the neurotransmitter into the presynaptic or surrounding cells via neurotransmitter transporters, and miRNAs may potentiate neurotransmission by inhibiting these transporters. miR-16 targets SERT, the serotonin transporter which terminates serotonergic signaling (Baudry et al., 2010). The selective serotonin reuptake inhibitor (SSRI) fluoxetine increases miR-16 and reduces SERT expression in the raphe nuclei, and miR-16 overexpression mimics the anti-depressant effects of fluoxetine in mouse models of depression. Alternatively, neurotransmission may be terminated by enzymes that degrade the neurotransmitter within the synaptic cleft, and miRNAs that target these enzymes are predicted to potentiate neurotransmission. For example, miR-132 inhibits synthesis of acetylcholinesterase (ACHE), the enzyme that catalyzes the hydrolysis of acetylcholine at the neuromuscular junction and in the central nervous system (Shaked et al., 2009; Shaltiel et al., 2013). In mice, stress paradigms elevate miR-132 levels in the hippocampus, reduce acetylcholinesterase levels, and impair performance in hippocampus-dependent tasks, such as the Morris water maze, suggesting that miR-132 may contribute to cognitive deficits following exposure to stressful stimuli by regulating ACHE (Shaltiel et al., 2013).

miRNAs also target mRNAs that encode enzymes and cytoskeletal proteins that influence receptor surface expression, internalization, and degradation. For example, miR-125a regulates PSD-95 synthesis in primary neurons (Muddashetty et al., 2011). PSD-95 directly interacts with AMPA receptor regulatory proteins to stabilize AMPA receptor surface expression (Yudowski et al., 2013). Changes in miR-125a activity are, therefore, expected to affect AMPA receptor surface levels, but this has yet to be verified. In addition, miR-146a-5p regulates the synthesis of dendritic microtubule-associated protein 1B (MAP1B), which regulates group 1 mGluR-induced AMPA receptor endocytosis (Chen and Shen, 2013). Overexpression of miR-146a-5p appears to block mGluR-dependent AMPA receptor endocytosis. When miR-146a-5p is inhibited, AMPA receptor endocytosis is enhanced without affecting total GluA1 protein levels, and synaptic transmission (measured by mEPSC frequency) is depressed by a MAP1B-dependent mechanism. It is feasible that miRNA-mediated inhibition of mRNAs encoding cell surface receptors provides greater specificity for targeting select receptor subunits or receptor types, whereas miRNA targeting of mRNAs that encode proteins like PSD-95 and MAP1B with the potential to interact with multiple surface receptors may provide a mechanism for coordinating the trafficking and surface levels of classes of receptors.

These studies demonstrate that miRNAs utilize diverse mechanisms to influence neurotransmitter signaling, yet many gaps remain in our knowledge in this research area. Many neurodegenerative and neuropsychiatric disorders are strongly associated with deficits in specific neurotransmitter systems. For example, Parkinson’s disease is associated with the loss of dopamine signaling, and depression therapies often target serotonin. miRNAs associated with disease-associated neurotransmitter systems may provide further insight into disease etiology and serve as novel therapeutic targets.

### miRNAs in Growth Factor Signal Transduction

miRNAs regulate growth factor signaling by a variety of mechanisms. Several miRNAs directly target the mRNAs that encode growth factors. The 3′UTR of *BDNF* mRNA, which encodes brain-derived neurotrophic factor (BDNF), appears to be particularly important for regulating stimulus-induced BDNF synthesis in neurons (Lau et al., 2010). miR-26a and miR-26b both target the 3′UTR of *BDNF* mRNA in HeLa cells (Caputo et al., 2011). miR-140 and miR-211 target the 3′UTR of *BDNF* in human astrocyte cultures and regulate their inflammation-induced proliferation (Tu et al., 2017; Zhang et al., 2017) Similarly, miR-206 directly targets the 3′UTR of mouse *Bdnf* mRNA and inhibits BDNF protein synthesis in Neuro2A cells (Lee et al., 2012). miR-206 is elevated in the brains of Alzheimer’s disease (AD) model mice and in the temporal cortex of AD patients, and inhibition of miR-206 in AD mice enhances BDNF and improves memory function. Dysregulation of miRNAs that target growth factors may, therefore, contribute to neurological disease and may provide a novel therapeutic target.

Some growth factors, such as the neuregulins, are synthesized as transmembrane precursors that undergo proteolytic processing to release a soluble extracellular signaling domain. Others, such as BDNF, are packaged into vesicles and released into the extracellular space via the secretory pathway. The role of miRNAs in these processes is currently unknown. Similarly, whether miRNAs also function in the termination of neuronal growth factor signaling has not been examined.

miRNAs also target mRNAs that encode growth factor receptors. miR-149 inhibits neuregulin signaling by targeting the mRNA that encodes the ErbB3 receptor (Bischoff et al., 2015). Different receptors for a single growth factor may have different, or even antagonistic, effects on cell function. For example, the TrkB receptor inhibits apoptosis in response to BDNF signaling, but the p75NTR BDNF receptor stimulates apoptosis. miRNA targeting of specific receptors can therefore dramatically affect cellular response to an extracellular signal. miR-592 levels decline in the mouse hippocampus following ischemic injury, allowing the rapid synthesis of its target p75NTR, which in turn promotes apoptotic signaling and neuronal death (Irmady et al., 2014). miRNAs targeting TrkB would be expected to have the opposite effect on neuronal health.

### miRNAs Influence Intrinsic Neuronal Excitability

miRNAs can regulate intrinsic neuronal excitability by targeting ion channels that are voltage-gated, rather than ligand-gated. miR-129 inhibits synthesis of Kv1.1 and miR-324 inhibits synthesis of Kv4.2, both of which are subunits of voltage-gated potassium channels that open in response to membrane depolarization and allow an outflux of potassium ions (Raab-Graham et al., 2006; Gross et al., 2016). These channels limit the frequency and propagation of action potentials, and the loss of either channel increases seizure susceptibility in mouse models (Rho et al., 1999; Barnwell et al., 2009). In mice, kainic acid-induced *status epilepticus* increases miR-324-5p association with RISC component Ago2 and recruits *Kcnd2* mRNA (which encodes Kv4.2) to the RISC (Gross et al., 2016). Inhibition of miR-324-5p delays seizure onset following kainic acid and reduces kainic acid-induced cell death in cultured hippocampal neurons and in the hippocampus *in vivo*. Furthermore, inhibition of miR-324-5p blocks kainic-acid induced downregulation of Kv4.2 in neurons *in vitro* and *in vivo*, and inhibition of miR-324-5p fails to delay seizure onset in *Kcnd2* knockout mice, suggesting that increased Kv4.2, and presumably reduced intrinsic excitability, contribute to the neuroprotective effects of inhibition of miR-324-5p. Several other miRNAs alter seizure susceptibility in mice or rats when inhibited or overexpressed briefly before or after pharmacologically induced seizures (Tiwari et al., 2018); however, none of these miRNAs were shown to target the mRNAs of ion channels or transporters, suggesting that they do not directly alter intrinsic excitability. Of note, a recent study demonstrated that miR-101, when inhibited briefly during early development in mice, increases neuronal excitability later in life (Lippi et al., 2016). One of the targets potentially mediating this effect is the chloride importer NKCC1, downregulation of which is important for the “switch” of GABAergic neurons from excitatory to inhibitory during development (Ben-Ari et al., 2012). This switch is essential to reduce spontaneous neuronal excitation during development. Loss of the repressive effect of miR-101 on NKCC1 during this critical period is expected to lead to a delay of this reduction in intrinsic neuronal excitability and may, therefore, contribute to the observed network changes and increased excitability later in life. These studies suggest that by regulating intrinsic excitability miRNAs not only regulate the likelihood that a neuron will respond to a given excitatory signal by firing an action potential but also regulate neuronal vulnerability to seizures and seizure-induced neuronal loss.

### miRNAs Influence Intracellular Signaling Cascades

Ion channels regulate neuronal signaling in part by regulating the membrane potential and the likelihood that the neuron will fire an action potential. By contrast, metabotropic neurotransmitter receptors (e.g., the mGluRs and other G-protein couple receptors) and growth factor receptors, signal through intracellular protein cascades mediated by kinases, phosphatases, and second messenger systems. miRNAs can regulate signaling responses to these ligands by targeting the mRNAs that encode these downstream protein components. miR-126 regulates phosphoinositide-3-kinase (PI3K) downstream of insulin-like growth factor 1 (IGF-1) by targeting the mRNAs that encode insulin receptor substrate 1(IRS-1) and the PI3K regulatory subunit p85β (Guo et al., 2008; Zhang et al., 2008; Ryu et al., 2011; Zhu et al., 2011; Kim W. et al., 2014). In neuroblastoma cell lines, overexpression of miR-126 impairs IGF-1 induced cell proliferation and increases sensitivity to the neurotoxic agent 6-OHDA, while inhibition of miR-126 enhances the protective effects of IGF-1 against cell death (Kim W. et al., 2014). Overexpression of miR-126 in primary neurons also reduces the neuroprotective effects of BDNF, nerve growth factor (NGF), and IGF-1 and increases neuronal vulnerability to amyloid beta (Aβ) induced neurotoxicity (Kim Y.-K. et al., 2016). Furthermore, overexpression of miR-126 reduces IGF-1-induced increases in Akt and p85β; both of which mediate PI3K signaling downstream of IGF-1 (Kim Y.-K. et al., 2016). Together, these studies demonstrate that miR-126 regulates multiple proteins downstream of growth factor signaling and regulates neuronal responses to multiple growth factors. Furthermore, miR-126 levels are increased in dopaminergic neurons in the brains of Parkinson’s disease patients, suggesting that dysregulation of miRNA activity may contribute to deficits in growth factor signaling and to neuronal cell death in neurodegenerative disease (Kim W. et al., 2014).

Within motor neuron axons, miR-183 directly targets the mRNA that encodes mTOR, an obligate component of mTORC1 and mTORC2 (mTOR Complexes 1 and 2), which regulate mRNA translation and the cytoskeletal architecture, respectively (Kye et al., 2014). In mouse models of spinal muscular atrophy (SMA), deficiency of SMN protein leads to an accumulation of miR-183 and other miRNAs in the axon, and axonal synthesis of mTOR protein is impaired, leading to reduced mTORC1 and mTORC2 signaling and reduced axon outgrowth. miRNA levels are also elevated in fibroblasts derived from SMA patients. Furthermore, inhibition of miR-183 in mouse models of SMA improves motor performance and rodent survival. Together, these data suggest that miRNA elevation may contribute to motor neuron deficits in SMA, and that inhibiting excess miRNA activity may have therapeutic utility.

Similarly, miR-128 regulates signaling downstream of dopamine 1 receptors (Drd1) in motor neurons by targeting several components of the ERK1/2 pathway (Tan et al., 2013). Loss of miR-128 in Drd1-positive neurons leads to motor hyperactivity and lethal seizures. An ERK1/2 inhibitor corrects ERK2 phosphorylation and motor hyperactivity in these mice, demonstrating a potential role of miR-128-regulated ERK1/2 signaling in controlling neuronal activity.

Neuronal signaling may be sensitive to changes in miRNA activity at every stage of signal transduction. Furthermore, single miRNAs may regulate multiple steps (as in the case of miR-128), potentially even within multiple signaling pathways. The examination of single miRNA targets may therefore be insufficient to predict the effects of miRNA activity on neuronal signaling, and the effects of individual miRNAs on neuronal signaling may differ across developmental stages, cell types, brain regions, and disease states. Further research is needed to elucidate the many roles of miRNAs in the regulation of neuronal signaling and to elucidate how miRNA dysregulation may contribute to signaling deficits in neurological disease.

## The Schizophrenia-Associated miRNA miR-137 Regulates Neuronal Signaling

In the following sections we will consider one specific miRNA, miR-137, that has been linked to the regulation of pre- and postsynaptic signaling as well as neuronal maturation and several forms of synaptic plasticity. More intriguingly, miR-137 dysregulation has been associated with intellectual disability, autism, bipolar disorder, and schizophrenia, suggesting that miR-137 is critical for human brain function.

### miR-137 and 1p21.3 Deletion Syndrome

Remarkably, miR-137 provided the first definitive evidence that dysregulation of a single miRNA can significantly impact human brain function. Animal studies had demonstrated that miRNAs were important for neuronal function, and human studies investigating 22q11DS suggested that broad dysregulation of the miRNA pathway contributes to neurodevelopmental disorders. However, prior to 2011 no mutations in miRNA genes had been associated with neurological or cognitive dysfunction.

Carter et al. (2011) identified a novel hemizygous microdeletion syndrome affecting the 1p21.3 region in 4 individuals from 3 families that presented with ASD and severe speech delay (Table 1). Two of the 4 patients also presented with intellectual disability. One patient carried a 10 kb deletion affecting only exon 6 of *DPYD*, which encodes dihydropyrimidine dehydrogenase, an enzyme involved in pyrimidine catabolism. At the time, the authors attributed the cognitive symptoms of 1p21.3 deletion patients to the loss of *DPYD*. However, loss-of-function mutations in *DPYD* had previously been described in human patients, and the *DPYD* mutation patients lacked the cognitive dysfunction noted in the 1p21.3 microdeletion patients (van Kuilenburg et al., 2010).

**Table 1:** 1p21.3 deletions are associated with intellectual disability and ASD in patients.

Reference	Carter et al., 2011				Willemsen et al., 2011					D’Angelo et al., 2015		Pinto et al., 2014	Tucci et al., 2016
Patient	1	2	3	4	1	2	3	4	5	1	2	8658_201	1
Age at Examination	13 years	6 years	7 years	11 years	42 years	38 years	?	33 years	18 years	15 years	8 years	?	10 years
Sex	Male	Female	Male	Male	Male	Male	Female	Male	Female	Female	Female	Female	Male
Intellectual Disability	+	+	–	–	+	+	+	+	+	+	+	–	+
Features of ASD	+	+	+	+	+	+	?	+	–	–	+	+	+
Speech Delay (+/-), Other Deficits	+	+	+	Regression	?	Deficient	?	Deficient	?	+	+	–	+
Motor Development Delay	–	+	–	–	?	?	?	?	?	+	+	?	+
Macrocephaly	–	–	+	–	–	–	?	–	–	+	+	–	–
Seizures (Age in Years)	Febrile (2)	–	–	–	–	–	?	–	–	–	–	–	Febrile (before 3)
Weight Percentile	?	75^th^	> 99^th^	25^th^	90^th^	> 98^th^	?	98^th^	> 98^th^	>97^th^	> 97^th^	Overweight	> 97^th^
1p Deletion Segment	21.3	21.3	21.3	21.3	21.3	21.3	21.3	21.3	21.3	22.1–21.2	21.3–13.3	21.3–21.2	21.3–21.1
*DPYD* Deletion	+	+	+	+ (exon 6)	+	+	+	+	+	+	+	–	–
*MIR137HG* Deletion	+	+	+	–	+	+	+	+	+	+	+	+	+
Other Notes	*PTCHD1* missense mutation		Sibling of patient 2	No *MIR137HG* deletion	Sibling of 2 and 3		No clinical exam					No *DPYD* deletion	No *DPYD* deletion


*This table summarizes the clinical features of patients with hemizygous deletions in the 1p21.3 region, which contains *MIR137HG* as well as protein-coding genes, e.g., *DPYD*. Most patients have mild to moderate intellectual disability and features of ASD. This deletion may also contribute to delays in language and/or motor development. A few patients have macrocephaly, and most patients are obese. Most patients have deletions that affect both *MIR137HG* and *DPYD*, but a few patients have deletions that affect only one of these genes. All of these patients exhibit features of ASD, but only one of the three has intellectual disability.*

Willemsen et al. (2011) then identified 5 additional 1p21.3 microdeletion patients, all with mild to moderate intellectual disability and 3 with features of ASD, and identified the genomic regions affected in each patient using genome-wide array analysis. Excluding the *DPYD* exon 6 deletion carrier (Carter et al., 2011), the shortest region of overlap present in all patients included *DPYD* but also *MIR137HG* (i.e., miR-137 “host gene”), which encodes a long-non-coding RNA that serves as the pri-miRNA for miR-137. Lymphoblastoid cell lines (LCLs) derived from the 1p21.3 microdeletion patients expressed significantly lower levels of mature miR-137 and increased expression of validated miR-137 targets (Willemsen et al., 2011). By contrast, plasma pyrimidine levels were unaffected, suggesting no deficiency in pyrimidine metabolism. Analysis of human brain tissue also revealed that miR-137 is highly expressed in the human cortex, hippocampus, and other brain regions critical for cognition (Willemsen et al., 2011).

Willemsen et al. (2011) concluded that the loss of miR-137 specifically contributes to the intellectual disability, ASD, and other cognitive phenotypes observed in 1p21.3 microdeletion patients, therein providing the first evidence that mutations, in this case a hemizygous deletion, in miRNA genes contribute to human cognitive function and dysfunction. To date, 12 patients with 9 independent 1p21.3 microdeletions affecting *MIR137HG* have been described in the literature (Tucci et al., 2016). The smallest region of overlap shared by all 9 microdeletions includes only *MIR137HG*, confirming that the loss of miR-137 underlies the shared cognitive phenotypes described in these patients.

Several patients carrying 1p21.3 duplications have also been described in the literature (Table 2) (Lo et al., 1998; Utkus et al., 1999; Mazzeu et al., 2007; Pinto et al., 2014; Brečević et al., 2015). Similar to 1p21.3 deletion carriers, the duplication patients have intellectual disability and most exhibit features of ASD. A similar convergence of phenotypes between duplication and deletion carriers has been observed for other loci. For example, 22q11.2 duplications and deletions increase ASD risk (Biswas and Furniss, 2016; Wenger et al., 2016). To our knowledge, miR-137 levels have not been examined in patients carrying 1p21.3 duplications, and patient phenotypes have not been attributed to any single gene in the 1p21.3 region. Further research is needed to determine if miR-137 overexpression contributes to cognitive deficits in these patients.

**Table 2:** 1p21.3 duplication is associated with intellectual disability and ASD in patients.

Reference	Lo et al., 1998	Utkus et al., 1999	Mazzeu et al., 2007	Brečević et al., 2015	Pinto et al., 2014	Piccione et al., 2010
Age at Examination	8 years	3 years	3 years	19 years	?	17 years
Sex	Male	Male	Female	Male	Male	Male
Intellectual Disability	Mild	Moderate	+	Moderate-severe	Mild	Mild
Features of ASD	?	?	?	+	+	+
Speech Deficits	?	No speech	No speech	Severe deficit	Delayed	+
Motor Development/Deficits	?	Delayed, hyperactivity	Delayed	Hyperactivity	–	+
Microcephaly	+	+	–	–	–	–
Seizures (Age in Years)	Tonic (7)	?	?	Febrile (2–3)	–	–
Segment of 1p Duplicated	22.1-13.1	22.1-13.3	31.1-13.3	21.3-21.2	21.3-21.2	21.2-13.3
*MIR137HG* Duplicated	+	+	+	+	+	–


*This table summarizes the clinical features of patients carrying duplications of the 1p21.3 region, which contains *MIR137HG* as well as protein-coding genes, e.g., *DPYD*. In addition to intellectual disability and ASD, the duplication may also contribute to delays in language and motor development. Several patients also have a history of seizures and/or microcephaly. However, these patients also carry duplications of adjoining regions (e.g., 1p21.2) that might influence the patient phenotype. Notably, a patient described by Piccione et al. (2010) also had mild intellectual disability and ASD but lacked duplication of the 1p21.3 region.*

Animal models might address the role of *MIR137HG* deletion and duplication without the confounding influence of neighboring genes. Siegert et al. (2015) found that miR-137 overexpression limited to the dentate gyrus (hippocampus) causes deficits in presynaptic plasticity and hippocampal memory formation. Dong et al. (2012) also generated transgenic mice that overexpress miR-137, but to our knowledge these mice have not undergone behavioral testing and altered coat color is their only documented phenotype. In theory, miR-137 overexpressing mice might be used to assess whether miR-137 contributes to cognitive deficits in 1p21.3 duplication carriers. Additional research is first needed to determine how 1p21.3 duplication affects miR-137 levels within human brain and to generate mice with appropriately tuned miR-137 expression.

miR-137 knockout is embryonic or early postnatal lethal in mice (Crowley et al., 2015; Cheng et al., 2018). Initially, Crowley et al. (2015) found that miR-137 levels in the brains of mice heterozygous for *Mir137* knockout do not differ significantly from wildtype mice, and heterozygous mice exhibit normal behavior by most measures. Recently, however, Cheng et al. (2018) generated mice in which conditional *Mir137* knockout was driven by the *Nestin* promoter (cKO). miR-137 levels were reduced by ∼50% in mice heterozygous for *Mir137* cKO (Cheng et al., 2018, and these mice exhibited ASD-like behaviors, including increased perseverative behaviors (e.g., increased self-grooming and marble burying) and impaired social behavior (e.g., in the three-chamber and social discrimination tests) (Cheng et al., 2018). In contrast to Crowley et al. (2015) and Cheng et al. (2018) found that germline heterozygous knockout of *Mir137* produced similar miR-137 loss and social deficits to the *Nestin*-driven cKO. Phenotypic differences may be due in part to the use of different mouse strains (C57Bl/6J vs. 129S6/SvEvTac). However, both studies are consistent with the hypothesis that miR-137 depletion within the brain contributes to ASD-like behaviors in mice and to ASD risk in 1p21.3 microdeletion patients. The next section will examine recent evidence suggesting a role for miR-137 loss-of-function in another psychiatric disease: schizophrenia.

### miR-137 and Schizophrenia

Schizophrenia is a debilitating psychological disorder with no cure, poorly understood etiology, and complex underlying genetics. The symptoms of schizophrenia usually emerge during adolescence or early adulthood and generally fall into three broad categories: positive, negative, and cognitive (Tandon et al., 2009). Positive symptoms include auditory and visual hallucinations, delusions, and other distortions of reality. Negative symptoms refer to the loss or blunting of conative and affective functions. Specific negative symptoms include anhedonia (the loss of pleasure), reduced social drive, and apathy. Cognitive symptoms vary widely but include disorganized thoughts and depression. Across patients, the symptoms of schizophrenia are highly heterogeneous, with different patients experiencing different combinations and severities of symptoms. Antipsychotic therapies generally only treat the positive symptoms. The lifetime prevalence of schizophrenia is approximately 0.7%, with an annual incidence of an average of 15 per 100,000 people-years (Tandon et al., 2008). Schizophrenia therefore poses a substantial global economic burden, with schizophrenia associated costs in the United States estimated at $60 billion in 2013 alone (Chong et al., 2016).

Twin studies suggest that schizophrenia is highly heritable (approximately 81% heritable) (Sullivan et al., 2003). Yet the dysfunction of no single gene can explain all symptoms of schizophrenia or the presence of schizophrenia in all patients; and rare, common, and *de novo* variants have all been linked to schizophrenia susceptibility. Furthermore, genetic variants that have been linked to schizophrenia often contribute to other neurodevelopmental or psychiatric disorders, including ASD, bipolar disorder, and intellectual disability (Sullivan et al., 2012).

In 2011, a genome-wide association study (GWAS) identified a novel schizophrenia-associated single nucleotide polymorphism (SNP) within *MIR137HG* (rs1625579) (Ripke et al., 2011) (Table 3). Of the other loci that reached genome-wide significance within either the schizophrenia data set or within a joint data set including bipolar disorder patients, four loci were associated with validated mRNA targets of miR-137: *TCF4, CACNA1C, CSMD1*, and *C10orf26* (Kwon et al., 2011; Ripke et al., 2011). *ZNF804A*, another miR-137 target, nearly met genome-wide significance in later studies (Kim et al., 2012; Ripke et al., 2013). Later GWAS studies replicated the association between schizophrenia and *MIR137HG* (Ripke et al., 2013, 2014). Most notably, in an analysis of 36,989 cases and 113,075 controls, Ripke et al. (2014) identified 108 schizophrenia associated loci that met genome-wide significance: a SNP within *MIR137HG* (rs1702294) showed the second strongest association with SCZ (Table 4). The minor allele at the schizophrenia associated loci (rs1625579 and rs1702294) appears to be protective against schizophrenia, and the risk allele is the major allele (Ripke et al., 2011, 2014).

**Table 3:** Evidence linking MIR137HG rs1622579 to schizophrenia.

SNP	Allele	Freq	SCZ	Associated with:	Sample/Population	Reference
rs1625579	G	Minor	Protective	• Increased miR-137 levels	Human fibroblast-derived neurons	Siegert et al., 2015
				• Reduced cortical surface area	Control subjects	Vogel et al., 2018
				• Greater reduction in volume of mid-posterior corpus callosum (relative to TT SCZ patients)	SCZ patients	Patel et al., 2015
				• Allele carriers with severe negative symptoms are more likely to have cognitive deficits	SCZ patients	Green et al., 2013
	T	Major	Risk^∗^	• Lower miR-137 levels in dorsolateral prefrontal cortex	Control subjects	Guella et al., 2013
				• Reduced white matter integrity	SCZ patients	Lett et al., 2013
				• Reduced hippocampal volume	SCZ patients	Lett et al., 2013
				• Larger left lateral ventricle volume	SCZ patients	Lett et al., 2013
				• Lower cortical surface area	First degree relatives of SCZ patients	Vogel et al., 2018
				• Differences in occipital, parietal and temporal lobe gray matter concentration	SCZ patients	Wright et al., 2016
				• Reduced functional anisotropy in fronto-striatal regions	SCZ patients	Kuswanto et al., 2015
				• Reduced functional anisotropy (whole brain)	First degree relatives of SCZ patients	Vogel et al., 2018
				• Increased functional connectivity between right dorsolateral prefrontal cortex and left hippocampal field relative to heterozygous subjects	Control subjects	Liu B. et al., 2014
				• Increased functional connectivity between right amygdala and cingulate and prefrontal cortex	Control subjects	Mothersill et al., 2014
				• Greater activation in posterior right medial frontal gyrus, BA6	Control and first/second degree relatives of SCZ or BPD patients	Whalley et al., 2012
				• Hyperactivation of the dorsolateral prefrontal cortex during working memory task	Control and SCZ patients	van Erp et al., 2014
				• Earlier age-of-onset of psychosis	SCZ patients	Lett et al., 2013
				• Reduced auditory P300 amplitude	SCZ patients	Decoster et al., 2012
				• Lower BADDS incongruence dimension scores	Patients only (SCZ and related disorders)	Cummings et al., 2013
				• Lower OPCRIT-derived positive symptom scores	Patients only (SCZ and related disorders)	Cummings et al., 2013
				• Worse performance in verbal episodic memory	Control and patients (SCZ and related)	Cummings et al., 2013
				• Worse performance in extradimensional set shifting	Control and patients (SCZ and related)	Cummings et al., 2013
				• Greater symptom severity (PANNS)	Female SCZ patients	Kandratsenka et al., 2018
				• Worse negative symptoms (PANNS)	SCZ patients	Kuswanto et al., 2015
				• Worse attention and processing speed in cognitive assessment	SCZ patients	Kuswanto et al., 2015
				• Worse working memory performance (BACS, digit sequencing task)	SCZ patients	Ma et al., 2014
	No difference in alleles:			• Total brain, gray matter, white matter, or hippocampal volume	Control subjects	Cousijn et al., 2014
				• IQ	Control and patients (SCZ and related)	Cummings et al., 2013
				• White matter microstructure	Control subjects	Kelly et al., 2014
				• Cortical thickness	Control and SCZ patients	Lett et al., 2013
				• Ventricle or hippocampal volume	Control and SCZ patients	Patel et al., 2015
				• Cortical thickness	Control and first degree relatives of SCZ patients	Vogel et al., 2018


*Risk identified by ^∗^Ripke et al. (2011). BADDS, Bipolar Affective Disorder Dimension Scale; OPCRIT, Operational Criteria Checklist for Psychotic Illness; PANNS, Positive and Negative Syndrome Scale; BACS, Brief Assessment of Cognition in Schizophrenia; BPD, bipolar disorder; Freq, allele frequency; SCZ, schizophrenia. This table summarizes the experimental evidence linking the GWAS SNP rs1622579 to schizophrenia risk and symptom severity. The SNP may also influence miR-137 levels in neurons, brain structure, brain activity, and cognition in human subjects with or without schizophrenia, as indicated.*

**Table 4:** Evidence linking additional MIR137HG SNPs to schizophrenia.

SNP	Allele	Freq	SCZ	Associated with:	Sample/Population	Reference
rs1198588	A	Minor	Protective	• Increased miR-137 levels (in combination with other protective SNPs)	Human fibroblast-derived neurons	Siegert et al., 2015
				• Increased miR-137 levels	hiPSC-derived neurons	Forrest et al., 2017
				• Increased accessibility of *MIR137HG* promoter open chromatin region	hiPSC-derived neurons	Forrest et al., 2017
				• Reduced dendritic branching and length	hiPSC-derived neurons	Forrest et al., 2017
				• Reduced GluA1-positive dendritic protrusions	hiPSC-derived neurons	Forrest et al., 2017
	T	Major	Risk^∗^	• Reduced functional anisotropy in fronto-striatal regions	SCZ patients	Kuswanto et al., 2015
				• Worse negative symptoms (PANNS)	SCZ patients	Kuswanto et al., 2015
rs2660304	G	Minor	Protective	• Increased miR-137 levels (GG vs. TT)	Human fibroblast-derived neurons	Siegert et al., 2015
				• No effect on miR-137 levels (GT vs TT)	hiPSC-derived neurons	Forrest et al., 2017
	T	Major	Risk^∗∗^	• Reduced promoter activity relative to G allele	SH-SY5Y cells	Warburton et al., 2016
rs1702294	T	Minor	Protective			
	C	Major	Risk^∗∗∗^	• Lower performance IQ and full-scale IQ	Control and patients (SCZ and related)	Cosgrove et al., 2017
				• Worse social cognition (lower scores in Hinting Task)	Control and patients (SCZ and related)	Cosgrove et al., 2017
				• Worse attentional control (increased reaction time in Sustained Attention to Response Task)	Control and patients (SCZ and related)	Cosgrove et al., 2017
1:g.98515539A > T	A	Major	Protective	• Higher miR-137 levels	hiPSC-derived neurons	Forrest et al., 2017
				• Increased accessibility of *MIR137HG* promoter open chromatin region	hiPSC-derived neurons	Forrest et al., 2017
				• Reduced dendritic branching and length	hiPSC-derived neurons	Forrest et al., 2017
				• May reduce GluA1-positive and PSD95-positive dendritic protrusions (trend, but not significant)	hiPSC-derived neurons	Forrest et al., 2017
	T	Rare	Risk^∗∗∗∗^	• Lower reporter gene transcription (in neuron-like cell line, but not in HeLa cells)	SH-SY5Y cells	Duan et al., 2014
				• Reduced transcription factor YY1 binding	SH-SY5Y cells, nuclear extracts	Duan et al., 2014



*Risk identified by ^∗^Ripke et al. (2013), ^∗∗^Goes et al. (2015), ^∗∗∗^Ripke et al. (2014), ^∗∗∗∗^Duan et al. (2014). PANNS, Positive and Negative Syndrome Scale; hiPSC, human induced pluripotent stem cell; Freq, allele frequency; SCZ, schizophrenia. This table summarizes the findings of recent studies examining three common schizophrenia-associated SNPs (rs1198588, rs2660304, and rs1702294) and one rare schizophrenia-associated SNP (1:g.98515539A > T). Like rs1625579, these SNPs appear to influence miR-137 levels, brain structure, brain activity, and cognition in human subjects with or without schizophrenia. Also like rs1625579, these SNPs may also influence symptom severity in schizophrenia patients. Note that for all SNPs except 1:g.98515539A > T the major (more common) allele is associated with increased schizophrenia risk, whereas the minor allele appears to be protective.*

Several studies suggest that *MIR137HG* alleles associated with increased schizophrenia risk are associated with lower miR-137 levels, whereas the protective allele is associated with higher miR-137 levels (Tables 3, 4). Guella et al. (2013) found that healthy individuals homozygous for the risk associated T allele at rs1625579 have lower levels of miR-137 in the dorsolateral prefrontal cortex relative to carriers of the protective G allele. However, no genotype-associated differences were observed in the tissue from schizophrenia patients. Warburton et al. (2016) recently found that rs1625579 is in strong linkage disequilibrium with a second SNP, rs2660304 that lies within the promoter region of *MIR137HG*. Reporter assays in SH-SY5Y cells demonstrate that the risk allele at rs2660304 inhibits transcription, suggesting by extension that the rs1625579 risk allele may be associated with reduced miR-137 synthesis. Currently, however, the evidence linking rs2660304 to schizophrenia is less robust than the for the loci identified by Ripke et al. (2011; 2013; 2014) (i.e., rs1625579, rs1198588, and rs1702294) (Sakamoto and Crowley, 2018).

Duan et al. (2014) identified a rare schizophrenia- and bipolar disorder-associated SNP (1:g.98515539A > T) in the enhancer element for *MIR137HG*. Reporter assays in neuroblastoma cell lines show that the risk allele reduces enhancer activity by greater than 50%. Correction of the risk (minor) allele to the major allele in human induced pluripotent stem cell (hiPSC)-derived neurons increases *MIR137HG* promoter accessibility, gene transcription, and miR-137 levels (Forrest et al., 2017). Similarly, correction of the risk allele to the minor (protective) allele at rs1198588 increases promotor accessibility, transcription, and miR-137 levels relative to neurons derived from isogenic lines carrying the risk allele (Forrest et al., 2017).

*MIR137HG* also contains a 15 nucleotide variable number tandem repeat (VNTR) near the 5′ end of the pre-miR-137 sequence (Table 5) (Bemis et al., 2008). The number of repeats ranges from 3 to 13 in humans, and higher numbers of repeats have been associated with lower levels of miR-137 in cell lines, cognitive deficits in healthy adults, and increased schizophrenia risk in patients (Bemis et al., 2008; Mamdani et al., 2013; Strazisar et al., 2014).

**Table 5:** A variable number tandem repeat (VNTR) in MIR137HG regulates miR-137 levels.

Allele	Frequency						
		
Repeats	A	B	C	D	Associated with:	Sample/Population	Reference
3	72%	77.07%	ND^∗^	ND^∗^	• Major allele^∗^, also shown as major allele in NCBI Reference Sequence NR_046105.1	Control/SCZ patients	Mamdani et al., 2013
						Control subjects, Sweden	Strazisar et al., 2014
4	9%	12.35%	74.16%	78%	• Associated with differences in Stroop facilitation, accuracy in congruent trials, and in total errors in Stroop test (relative to alleles with greater number of repeats)	Control subjects, Colombia	González-Giraldo et al., 2016
					• Lower miR-137 levels relative to 3 repeats	SH-SY5Y cells	Strazisar et al., 2014
5	<5%	4.61%	6.18%	8.30%			
6	<5%	2.47%	9.55%	4.80%			
7	<5%	1.64%	1.69%	4.10%			
8	<5%	0.65%	2.81%	2.40%	• More frequently found in SCZ patients than in control subjects	Control/SCZ patients	Strazisar et al., 2014
					• Lower miR-137 levels relative to 3 repeats	SH-SY5Y cells	Strazisar et al., 2014
9	6%	1.20%	1.69%	1.70%	• Lower miR-137 levels relative to 3 repeats	HEK293 cells	Mamdani et al., 2013
10	<5%	ND	2.81%	0.70%			
11	ND	ND	0.56%	ND			
12	ND	ND	0.56%	ND	• Lower pri-miR-137 processing and miR-137 levels relative to 3 repeats	A375 cells	Bemis et al., 2008
					• May differentially impact gene transcription relative to 4 repeats	SH-SY5Y cells	Warburton et al., 2015
13	<5%	ND	ND	ND	• Lower miR-137 levels relative to 3 repeats	HEK293 cells	Mamdani et al., 2013



*Allele Frequency References; Mamdani et al., 2013 (A), Strazisar et al., 2014 (B, control subjects only), Warburton et al., 2015 (C), González-Giraldo et al., 2016 (D). ND, not detected. ^∗^Bemis et al. (2008); Mamdani et al. (2013), and Strazisar et al. (2014) report that the 3 repeat allele as the major allele, while references C and D report the 4 repeat allele as the major allele. This table summarizes the findings of studies examining a 15 nucleotide VNTR that lies upstream (5′) of the pre-miR-137 sequence. The number of repeats in the VNTR regulates miR-137 levels in cell lines and may influence human cognition and schizophrenia risk.*

All of the above studies suggest that miR-137 loss-of-function increases schizophrenia risk. However, Siegert et al. (2015) reported significantly increased miR-137 levels in fibroblast-derived neurons from individuals with minor alleles at 4 schizophrenia-associated SNPs, including rs1625579 and rs2660304. The authors initially suggested that miR-137 gain-of-function may contribute to schizophrenia. However, the authors had identified the minor allele as the risk-associated allele, and existing evidence suggests the minor allele is associated with reduced schizophrenia risk relative to the major allele at these loci (Ripke et al., 2011). The authors later issued an Addendum recognizing that the presentation of their results had caused confusion among readers (Siegert et al., 2016). As noted by a recent review (Sakamoto and Crowley, 2018), the Siegert et al. (2015) data are actually in agreement with other studies that suggest that the risk alleles are associated with reduced miR-137 levels relative to the protective allele. However, to our knowledge, no known 1p21.3 microdeletion patient has shown signs of psychosis or been diagnosed with schizophrenia, and we would predict based on the findings of Willemsen et al. (2011) and Cheng et al. (2018) that these patients experience a significant reduction in brain miR-137 levels. Further research is needed to determine how schizophrenia-associated variation in *MIR137HG* affects miR-137 levels in human brain and to determine how this variation contributes to schizophrenia, ASD, and other psychiatric disorders.

### miR-137 in Neuronal Signaling

**FIGURE 4:**
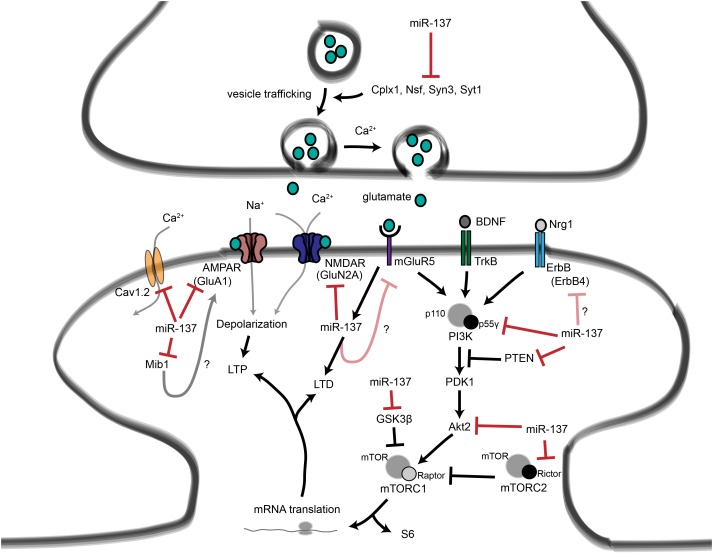
Proposed roles for miR-137 at the glutamatergic synapse. This figure summarizes the findings of Kwon et al. (2011); Zhao et al. (2013), Olde Loohuis et al. (2015); Siegert et al. (2015), and Thomas et al. (2017). miR-137 regulates presynaptic signaling by regulating vesicle trafficking in the axon terminal. miR-137 also targets the mRNA that encodes the L-type calcium channel subunit Cav1.2, which has also been linked to schizophrenia with genome-wide significance. Postsynaptically, miR-137 regulates the levels of glutamatergic receptor subunits GluA1 and GluN2A, and bioinformatic predictions suggest that miR-137 may target the metabotropic glutamate receptor mGluR5 as well as ErbB4, which regulates the strength of glutamatergic synapses. mGluR5 signaling, in turn, increases miR-137 levels. miR-137 also regulates proteins within the PI3K-Akt-mTOR pathway, e.g., p55γ, to regulate neuronal responses to BDNF and Nrg1 signaling. We propose that miR-137 may regulate PI3K-Akt-mTOR signaling downstream of mGluR receptors as well.

The mechanism by which miR-137 dysregulation might contribute to schizophrenia risk remains unclear. However, recent research suggests that miR-137 may play a particularly important role in regulating neuronal signaling (Figure 4), neuronal maturation, and synaptic plasticity. All of which are likely to be dysregulated in schizophrenia.

The first mechanism by which miR-137 regulates neuronal signaling is by targeting proteins essential for the release of neurotransmitter from the axon terminal (Siegert et al., 2015). Neurotransmitters are packaged into presynaptic vesicles within the axon terminal and released in response to action potential-induced influx of calcium. Siegert et al. (2015) validated the mRNAs that encode complexin-1 (Cplx1), *N*-ethylmaleimide-sensitive fusion protein (Nsf), synapsin-3 (Syn3), and synaptotagmin-1 (Syt1) as bona fide targets of miR-137. Overexpression of miR-137 reduces the levels of these proteins in the mossy fiber–CA3 pathway, reduces the number of vesicles in the active zone of the mossy fiber presynaptic terminal *in vivo*, and reduces the amplitude of the response evoked in mossy fiber-CA3 synapses by sustained low frequency stimulation of dentate granule cells. Overexpression of miR-137 in the dentate gyrus also impairs presynaptic LTP, a form of synaptic plasticity that involves increased neurotransmitter release from the mossy fiber axon terminal, and impairs hippocampus-dependent learning. By contrast, inhibition of miR-137 increases target protein levels, increases vesicle number in the active zone, and increases the induction of mossy fiber LTP. These results suggest that miR-137 plays a critical role in regulating presynaptic vesicle dynamics, thereby regulating synaptic signal transduction and synaptic plasticity.

A recent *in vitro* study confirmed a role of miR-137 in regulating vesicle numbers and subcellular location (He et al., 2018). This study also suggested a role of miR-137 in suppressing synapse formation by controlling the levels of proteins important for synaptogenesis. However, this study found that miR-137 overexpression had no effect on Syt1, Nsf, or Cplx1 protein levels in rat primary hippocampal neurons (He et al., 2018). Discrepancies between the findings of Siegert et al. (2015) and He et al. (2018) may be due in part to differences between the experimental systems employed (e.g., *in vivo* versus *in vitro* miR-137 overexpression), but further studies are required to clarify the role of miR-137 in the presynaptic compartment.

miR-137 regulates postsynaptic signaling by several mechanisms. miR-137 targets mRNA encoding the NMDA receptor subunit GluN2A and inhibits GluN2A synthesis in PC12 cells (Zhao et al., 2013). In *Drosophila*, miR-137 regulates the levels of NMDAR2 mRNA, which is homologous to mammalian GluN2A, as well as the mRNAs for the GABA receptors GABA-B-R3 and GABA-B-R1 and the dopamine receptor D2R (Kong et al., 2015). Furthermore, miR-137 levels are upregulated in Parkinson’s disease model flies and these mRNA targets are significantly downregulated, suggesting that miR-137 may contribute to signaling defects in this model system.

miR-137 also regulates glutamatergic signaling by targeting *Gria1*, which encodes AMPA receptor subunit GluA1 (Olde Loohuis et al., 2015; Thomas et al., 2017). mGluR5 signaling transiently increases miR-137 levels in rat primary hippocampal neurons, which inhibits AMPAR signaling and allows mGluR-dependent long term depression (LTD) at CA3-CA1 synapses (Olde Loohuis et al., 2015). Conversely, inhibition of endogenous miR-137 inhibits mGluR-dependent LTD expression, suggesting that miR-137 is necessary for this form of synaptic plasticity.

Overexpression of miR-137 in newly formed neurons in the dentate gyrus inhibits dendritic outgrowth *in vivo*, while inhibition of miR-137 enhances outgrowth (Smrt et al., 2010). Overexpression of mindbomb 1 (Mib1), a direct target of miR-137, blocks the effects of miR-137 overexpression on dendritic outgrowth, suggesting that miR-137 regulates neuronal maturation, in part, by regulating Mib1. Mib1 is an E3 ubiquitin ligase that regulates late phase LTP as well as learning and memory (Yoon et al., 2012). In *Drosophila*, Mib1 also positively regulates the synaptic localization of GluRIIA, GluRIIB, and GluRIIC receptors, which are homologues of the mammalian AMPA receptor subunits (Sturgeon et al., 2016), thus providing an another potential link between miR-137 and glutamatergic signaling.

A wide array of evidence suggests that deficits in glutamatergic signaling contribute to schizophrenia. Most of these deficits have been attributed to dysregulation of NMDA receptors (Coyle, 2006), and the NMDA receptor hypofunction hypothesis of schizophrenia originally arose from the observation that NMDA receptor antagonists, e.g., PCP and ketamine, mimic the positive and negative symptoms of schizophrenia (Luby et al., 1959; Itil et al., 1967; Krystal et al., 1994). SNPs in *GRIA1* and *GRIN2A* are also associated with schizophrenia with genome-wide significance (Ripke et al., 2014). miR-137’s ability to target multiple mRNAs associated with glutamatergic signaling suggest that dysregulation of miR-137 might contribute to glutamatergic signaling deficits in patients.

miR-137 may also regulate intrinsic neuronal excitability by targeting mRNAs that encode ion channels. For example, miR-137 targets *CACNA1C*, which encodes the L-type voltage-gated calcium channel subunit Cav1.2 (Kwon et al., 2011). Like *MIR137, CACNA1C* has been repeatedly linked to schizophrenia and bipolar disorder with genome-wide significance (Ripke et al., 2011, 2014; Smoller, 2013; Guan et al., 2014). Cav1.2 localizes to both the pre- and postsynaptic compartments of neurons within the rat hippocampus *in vivo* (Tippens et al., 2008). Selective inactivation of *CACNA1C* within the mouse neocortex and hippocampus impairs spatial memory and NMDA receptor-independent late phase LTP at Schaffer collateral-CA1 synapses, as well as inhibits MAPK signaling and CREB-induced transcription within the hippocampus (Moosmang et al., 2005). Within the nucleus accumbens, Cav1.2 also activates CamKII, which phosphorylates and stabilizes GluA1-containing AMPA receptors at the cell surface (Schierberl et al., 2011). These findings suggest that Cav1.2 dysregulation might contribute to miR-137-dependent neuronal phenotypes, but the relationship between miR-137 and Cav1.2 has not been examined within neuronal model systems.

miR-137 also targets intracellular proteins that mediate signaling events downstream of receptor activation. We recently reported that inhibition of miR-137 disrupts neuregulin 1α (Nrg1α) and BDNF signal transduction in mouse primary hippocampal neurons (Thomas et al., 2017). BDNF signaling is implicated in a wide range of neurodevelopmental and psychiatric disorders, including major depression, Rett syndrome, and addiction (Autry and Monteggia, 2012). Similarly, Nrg signaling has been repeatedly linked to psychiatric disorders, including schizophrenia, depression, and bipolar disorder (Mei and Nave, 2014).

Of note, we found that miR-137 regulates the levels of key proteins within the PI3K-Akt-mTOR pathway, specifically p55γ (a PI3K regulatory subunit), PTEN, Akt2, GSK3β, rictor, and mTOR, which mediate signaling downstream of Nrg1 and BDNF. The PI3K-Akt-mTOR pathway plays a critical role in multiple aspects of neuronal development and function, including cortical lamination, neurite outgrowth, dendritic spine development, synaptic plasticity, and learning and memory (Gross and Bassell, 2014; Crino, 2016). Furthermore, defects in PI3K-Akt-mTOR signaling underlie some forms of intellectual disability and autism and may contribute to schizophrenia (Law et al., 2012; Gross and Bassell, 2014; Crino, 2016).

We found that inhibition of miR-137 blocks Nrg1α-induced increases in phospho-S6, mRNA translation, and GluA1 synthesis in the dendrites of mouse primary hippocampal neurons and also blocks Nrg1α-induced dendritic outgrowth. Furthermore, inhibition of miR-137 blocks mTORC1-dependent responses to BDNF, specifically mRNA translation and dendritic outgrowth, while leaving mTORC1-independent S6 phosphorylation intact. By contrast, we found no evidence that Nrg1α or BDNF signaling regulates miR-137 activity. In summary, miR-137 regulates responses to multiple signaling ligands but may selectively regulate the PI3K-Akt-mTOR branch of Nrg and neurotrophin signaling.

Brain-derived neurotrophic factor may play a role in schizophrenia etiology, but whether BDNF plays a causative or merely incidental role remains unknown (Autry and Monteggia, 2012). Of these pathways, schizophrenia appears to have to strongest link to the Nrg/ErbB pathway, and SNPs in several of the genes encoding Nrg (*NRG1, NRG2, NRG3*, and *NRG6)* and all of the genes encoding ErbB receptors (*EGFR, ERBB2, ERBB3*, and *ERBB4*) have been linked to schizophrenia (Mei and Nave, 2014). Nrg1α-induced PI3K signaling is also impaired in lymphoblastoid cell lines derived from schizophrenia patients (Law et al., 2012), suggesting PI3K-Akt-mTOR signaling in response to Nrg1 may be impaired in schizophrenia in a manner that mirrors the effects of reduced miR-137.

miR-137 has also been linked to glucocorticoid signaling. In rat primary cortical neurons, miR-137 inhibits mRNAs, such as *Cox2, Dusp1, Dusp4, Egr2*, and *Sgk1*, that encode proteins involved in glucocorticoid receptor signaling, i.e., cyclooxygenase 2, dual specificity phosphatase 1 and 4, early growth response 2, and serum/glucocorticoid regulated kinase 1 (Vallès et al., 2014). Together with several previously validated miR-137 targets (*Cacna1c, Tcf4*, and *Znf804a*), these proteins may form a miR-137 target protein network that regulates glucocorticoid signaling. Whether altered miR-137 activity affects glucocorticoid signal transduction remains unknown, however.

The potential importance of miR-137-mediated regulation of glucocorticoid signaling has been corroborated by studies investigating environmental factors contributing to schizophrenia. Glucocorticoid signaling is a crucial component of the stress response. Though highly heritable, schizophrenia risk has also been linked to environmental factors. Many of these are maternal exposures that occur during the second trimester of gestation, which coincides with critical events in fetal brain development, including the production of the majority of the brain’s neurons, the migration of neurons within the cerebral cortex, and the formation of the thalamocortical projections (Koenig et al., 2002; Stiles and Jernigan, 2010). These schizophrenia-associated environmental exposures range from infections (e.g., influenza), to war (e.g., the German invasion of the Netherlands in 1940), to severe emotional stress (e.g., the loss of a husband) (Koenig et al., 2002). The common feature shared by these exposures is the triggering of the maternal stress response and the elevation of glucocorticoid signaling in both the mother and the developing fetus, which is hypothesized to disrupt the development of the hypothalamic-pituitary-adrenal (HPA) axis in the fetus (Gitau et al., 1998; Glover et al., 2009; Kinsella and Monk, 2009). These HPA axis disruptions are hypothesized to mediate the effects of stress on schizophrenia risk and underlie HPA axis disruptions in adult schizophrenia patients. The presence of multiple miR-137 targets within the glucocorticoid pathway suggests that disruption of miR-137 might lead to dysregulated synthesis of proteins that mediate glucocorticoid signaling and might, as a result, influence an individual’s susceptibility to schizophrenia associated environmental stressors.

These studies suggest that at a single synapse miR-137 might regulate presynaptic vesicle release, the availability of receptors at the cell surface, downstream signaling, and the induction of synaptic plasticity. These results also suggest that dysregulation of miR-137 might contribute to schizophrenia etiology by disrupting neuronal signaling and, consequently, neurodevelopment and synaptic plasticity.

## Conclusion/Future Directions

Traditionally, miRNA studies have followed a simple pattern. A miRNA or target of interest is identified and sequence information is used to predict a miRNA-target interaction. That interaction is then experimentally validated, and the function of the protein encoded by the mRNA is used to tie the miRNA to a cellular function.

The basic premise is quite true: a miRNA’s primary cellular function is to bind to larger nucleic acids. miRNAs have many targets though, and mounting evidence suggests miRNA interaction with an individual mRNA target site is dependent on the cell type in question, the developmental context of the cell, and the status of a plethora of intracellular and extracellular signaling events. miRNA-target interactions are highly dynamic and highly specific to the cellular context. As our understanding of these processes expands, we may discover that determining the function of a miRNA based on a single target is similar to judging the plot of an entire movie based on a single frame.

Conversely, natural environmental stimuli lead to widespread changes in miRNA profiles, rather than causing isolated changes in the activity of a single miRNA, and multiple miRNAs may influence the same signaling pathway. These miRNAs might act synergistically to promote dynamic shifts in neuronal signaling or antagonistically in order to stabilize neuronal signaling. In this review, we have focused on the roles of isolated miRNAs in neuronal signaling because most research to date has focused on the roles of isolated miRNAs rather than considering the ways that miRNAs might interact to tune neuronal signaling. We consider this a limitation of the field that should be addressed by future research.

miRNA research has also depended heavily on animal models, particularly rodents. However, recent evidence suggests that hundreds of human miRNAs do not exist in rodent models, and many miRNAs are primate- or even human-specific (Londin et al., 2015). Even well-conserved miRNAs can display dramatic differences across species in expression levels within the brain (Sousa et al., 2017). Furthermore, the emergence of new miRNAs may have been a critical factor in shaping the trajectory of human neocortical evolution (Kosik and Nowakowski, 2018). Human model systems are required in order to determine the functions of these human-specific miRNAs as well as to map their interactions with mRNA targets in cell types of interest. Recent advances in human pluripotent stem cell-based technologies, such as human brain organoids, may provide further insight into the unique roles of miRNAs in human brain function (Lancaster and Knoblich, 2014; Kelava and Lancaster, 2016).

miR-137 provides a particularly compelling example of the pivotal role that a single miRNA can have within the human brain. Loss of miR-137 underlies neurological symptoms in humans. Genetic variants that contribute to schizophrenia risk influence miR-137 levels in model systems. But perhaps most strikingly, miR-137 targets mRNAs encoded by genes that have been independently linked to schizophrenia and targets multiple components within multiple neuronal signaling pathways that have also been linked to schizophrenia. While miR-137 dysregulation almost certainly does not underlie every case of schizophrenia, the multifarious functions of miR-137 within neurons hint that miR-137 may be a missing link, tying together multiple signaling pathways and molecular mechanisms underlying schizophrenia etiology.

However, miR-137 is only one of many miRNAs that is critical for neuronal function and one of several miRNAs that may contribute to schizophrenia. Hauberg et al. (2016) found that genes associated with schizophrenia are more likely to encode targets of miRNAs and that schizophrenia associated genes are most enriched within the predicted target sets for miR-9, miR-485-5p, and miR-137, respectively. One possibility is that schizophrenia-associated miRNAs act synergistically to mediate disease risk. Loss-of-function in one miRNA might not significantly disrupt neuronal activity, while loss-of-function in multiple miRNAs leads to psychiatric disease. In this model, loss-of-function in miR-137 (e.g., due to the presence of risk-associated SNPs) might not be sufficient to cause disease but could increase vulnerability to further miRNA disruptions. Notably, 22q11DS is associated with the reduced synthesis of multiple miRNAs and high incidence of schizophrenia. To our knowledge no reports have linked the loss of miR-137 to neurological symptoms in 22q11DS patients or to relevant phenotypes in 22q11DS rodent models. Further research is needed to understand how miR-137 interacts with other schizophrenia risk factors, including other schizophrenia-associated miRNAs, to elucidate its role in disease etiology.

Perhaps the most valuable lesson from miR-137 research is this: human, disease-associated variants in miRNA genes can point researchers toward miRNAs that are critical for brain function. As genomic research continues to advance, we predict it will lead to the discovery of additional, equally interesting miRNAs, each seemingly endowed with the ability to regulate a biologically coherent network of targets having concerted effects on neuronal function. A better understanding of the links between individual targets and downstream effects will continue to inform our understanding of mechanisms of neurological diseases.

Manipulation of miRNAs has also emerged as a pharmacologic strategy for neuroprotection and restoration of neural function. The well-documented dysregulation of miRNAs in neurological disorders, combined with the ability of a single miRNA to regulate the expression of multiple disease-associated genes makes miRNAs intriguing as therapeutic targets (Chan and Kocerha, 2012). In recent years, many preclinical studies utilizing animal models have evaluated the therapeutic utility of miRNA mimics and antimiRs, which simulate or inhibit miRNA activity, respectively (Chan and Kocerha, 2012; Chakraborty et al., 2017; Rupaimoole and Slack, 2017). Multiple miRNA-based therapeutics have entered Phase I and Phase II clinical trials for the treatment of several forms of cancer as well as type 2 diabetes and hepatitis C (Rupaimoole and Slack, 2017). As our understanding of miRNAs within the human brain continues to expand, we predict that miRNA-based therapeutics targeting neurological diseases will reach patients as well.

## Author’s Note

The content of this review was adapted from KTT’s dissertation (Thomas, 2017).

## Author Contributions

GB and KT contributed to the conceptualization and design. KT wrote the original draft. KT, CG, and GB performed the writing, review, and editing of the manuscript.

## Conflict of Interest Statement

GB and CG are co-inventors on US patent 9,932,585 B2. The remaining author declares that the research was conducted in the absence of any commercial or financial relationships that could be construed as a potential conflict of interest.
